# Systematic review: outcomes and adverse events from randomised trials in Crohn's disease

**DOI:** 10.1111/apt.15174

**Published:** 2019-03-03

**Authors:** Heather Catt, Dyfrig Hughes, Jamie J. Kirkham, Keith Bodger

**Affiliations:** ^1^ Department of Biostatistics University of Liverpool Liverpool UK; ^2^ Centre for Health Economics and Medicines Evaluation Bangor University Bangor UK; ^3^ Digestive Diseases Centre Aintree University Hospital NHS Trust Liverpool UK

## Abstract

**Background:**

The suitability of disease activity indices has been challenged, with growing interest in objective measures of inflammation.

**Aim:**

To undertake a systematic review of efficacy and safety outcomes in placebo‐controlled randomised controlled trials (RCTs) of patients with Crohn's disease.

**Methods:**

MEDLINE, EMBASE, CINAHL and Cochrane Library were searched until November 2015, for RCTs of adult Crohn's disease patients treated with medical or surgical therapies. Data on efficacy and safety outcomes, end‐point definitions, and measurement instruments were extracted and stratified by publication date (pre‐2009 and 2009 onwards).

**Results:**

One hundred and eighty‐one RCTs (110 induction and 71 maintenance) were identified, including 23 850 patients. About 92.3% reported clinical efficacy endpoints. The Crohn's Disease Activity Index (CDAI) dominated, defining clinical response or remission in 63.5% of trials (35 definitions of response or remission). CDAI < 150 was the commonest endpoint, but reporting reduced between periods (46.4%‐41.1%), whilst use of CDAI100 increased (16.8%‐30.4%). Fistula studies most commonly reported fistula closure (9, 90.0%). Reporting of biomarker, endoscopy and histology endpoints increased overall (33.3%‐40.6%, 14.4%‐30.4% and 3.2%‐12.5%, respectively), but were heterogeneous and rarely reported in fistula trials. Patient‐reported outcome measures were reported in 41.4% of trials and safety endpoints in 35.4%. Many of the common adverse events relate to disease exacerbation or treatment failure.

**Conclusions:**

Trial endpoints vary across studies, over time and are distinct in fistula studies. Despite growth in reporting of objective measures of inflammation and in patient‐reported outcome measures, there is a lack of standardisation. This confirms the need for a core outcome set for comparative effectiveness research in Crohn's disease.

## 1 INTRODUCTION

Defining the key outcomes of therapeutic interventions and the best way to measure those outcomes is essential for clinical and regulatory decision‐making. Due to the complexity of Crohn's disease and the multitude of treatments, a number of different outcomes and outcome measures have been reported in clinical trials including symptom scores, composite disease activity indices and quality of life questionnaires.^1^, ^2^ Decision‐making also relies on the availability of good information on the unintended effects (harms) from treatments.

Heterogeneity in reporting of outcomes or measurement instruments within clinical trials may hinder the comparison of results within systematic reviews and inhibit the meaningful interpretation of individual studies.^3^ One way to mitigate this problem is the introduction of an agreed minimum set of standardised outcomes, to be measured and reported in all trials for a particular condition, referred to as a core outcome set.^4^ There is no core outcome set for Crohn's disease, although a model has been proposed for classifying outcomes for all inflammatory bowel diseases using the World Health Organisation International Classification of Functioning, Disability and Health (ICF).^5^ Recently, the International Consortium for Health Outcomes Measurement developed a “Standard Set” for inflammatory bowel disease with recommendations for the pragmatic measurement of outcomes in routine care to support benchmarking.^6^ Also recently published is a study protocol for the development of a core outcome set for inflammatory bowel disease^7^ and a core outcome set for fistulising Crohn's disease,^8^ indicating the importance of this research area. Future trial design and core outcome set development for Crohn's disease would benefit from a systematic synthesis of outcome reporting across published clinical trials, incorporating statistical testing and consideration of adverse events.

In this study, we systematically reviewed the literature to extract data on the outcomes and measurement instruments used, and the safety outcomes reported, in randomised clinical trials (RCTs) of treatments for Crohn's disease. Our aims were to explore the extent of heterogeneity among existing trials, to examine time trends in reporting and to generate insights to support future trial design and core outcome set development. Our results extend beyond the recently published literature in this area by including a broader set of interventions, offering statistical testing of time trends in outcome reporting and bringing new evidence on harms reporting in Crohn's disease.^8^, ^9^

## 2 METHODS

### 2.1 Systematic search

We registered review protocols with the International Prospective Register of Systematic Reviews (PROSPERO) database (CRD42016027656 http://www.crd.york.ac.uk/PROSPERO) and the Core Outcome Measures in Effectiveness Trials (COMET) database (http://www.comet-initiative.org/studies/details/867).

We conducted a systematic electronic search of the Cochrane Register of Controlled Trials (CENTRAL), EMBASE, MEDLINE and the Cumulative Index to Nursing and Allied Health Literature (CINAHL) until November 2015, with no date limits. The disease term “Crohn's disease” and the key word “outcome” were used. See Tables S1 to S4 for detailed search criteria.

### 2.2 Eligibility criteria and study selection

Randomised control trials of drug therapies (corticosteroids, 5‐ASAs, immunosuppressants, biologics and antibiotics), surgery and nondrug therapies (enteral nutrition, complementary and alternative medicine, probiotics and prebiotics) were included, as were RCTs of treatments for complications (strictures, fissures, abscesses and perforations). Eligible trials were conducted in adult patients (aged 18 or over) with Crohn's disease. Studies of inflammatory bowel disease populations were eligible provided outcomes were reported separately for Crohn's disease. Studies had to be published as full text in English.

Duplicates were removed after a complete list of RCTs was generated. Two reviewers (HC and JK) independently assessed the sample of 100 studies against eligibility criteria at the title and abstract screening stages and resolved discrepancies by discussion. A random sample of 100 was selected for review due to time constraints. The sample was generated by assigning each article a number and using a random number generator. There were no issues found when screening the 100 articles and the primary researcher (HC) screened the remaining papers independently. Full copies were obtained of all potentially eligible studies and reassessed against eligibility criteria by the primary researcher (HC). Reference was made to the second reviewer (JK) where needed.

### 2.3 Data collection

Data were extracted from the studies by the primary researcher. A randomly generated sample of 10 studies were reviewed and data extracted by the primary researcher and the secondary researcher (JK) checked the extraction. No inaccuracies were found in the data extraction of the sample of 10 papers and the primary researcher extracted data from the remaining papers independently. Studies were categorised as induction or maintenance with subcategories of medical vs surgical induction and maintenance of medically induced vs surgically induced remission. RCTs focusing solely on patients with fistulising disease were flagged to identify differences in reported outcomes. Efficacy and safety outcomes were recorded as reported as primary or secondary outcomes, or not specified as either. The efficacy outcomes were categorised in line with the method used by Ma et al^10^ as clinical or composite‐clinical, endoscopic, histologic, biomarkers and patient‐reported outcomes (PROs). Safety‐related outcomes were recorded as primary or secondary outcomes.

Adverse event reporting was recorded in specific categories: adverse events, serious adverse events, treatment‐related adverse events, treatment‐related serious adverse events, study withdrawal, abnormal laboratory results and adverse events by preferred term according to the Medical Dictionary for Regulatory Activities (MedDRA).^11^ Study withdrawals were categorised as due to adverse events, serious adverse events, treatment‐related adverse events, treatment‐related serious adverse events, treatment failure (insufficient therapeutic effect, exacerbation of Crohn's disease, development of complications or need for additional therapy, surgery or hospitalisation) or other reasons (protocol noncompliance, lost to follow‐up, prohibited medicine use or withdrawal of consent).

A critique of the methodological quality of the studies was unnecessary, as this project did not involve synthesis of outcome data.

### 2.4 Synthesis of results and analysis

A comprehensive record of efficacy and safety outcomes was generated and organised by outcome type. Our main analysis of efficacy outcomes focused on those designated as primary or secondary endpoints. We adopted a similar approach for safety‐related outcomes but also analysed all reported data for adverse events and study withdrawals. Adverse event reporting was considered at two levels of the MedDRA hierarchy: system organ classification (SOC) and higher level group term, the latter of which is considered a clinically relevant grouping of MedDRA preferred terms.^11^ Adverse events were grouped by MedDRA higher level group terms and ranked in the order of frequency of reporting. The top 10 ranked higher level group term adverse events were compared by trial type and drug class.

A secondary analysis considered the reporting of outcomes were not specified as primary or secondary endpoints. To mirror the increased focus on the importance of mucosal healing,^12^ the number of studies that reported additional endoscopic or histologic outcomes or the faecal calprotectin biomarker was assessed.

The proportion of studies reporting each type of outcome was calculated, by trial type. The results were stratified by into pre‐2009 and 2009 onwards and the changes over time in reporting were summarised in matrix form with outcome categories listed in rows and frequency of outcome reporting plotted in greyscale on a time axis.^10^ The statistical significance of any changes between time periods in outcome reporting was tested using the chi‐squared test (with 1 *df*, the critical value of chi is 3.84).

The review was reported in line with the Preferred Reporting Items for Systematic Reviews and Meta‐Analyses (PRISMA) statement and harms the checklist.^13^, ^14^

## 3 RESULTS

### 3.1 Systematic search results

**Figure 1:**
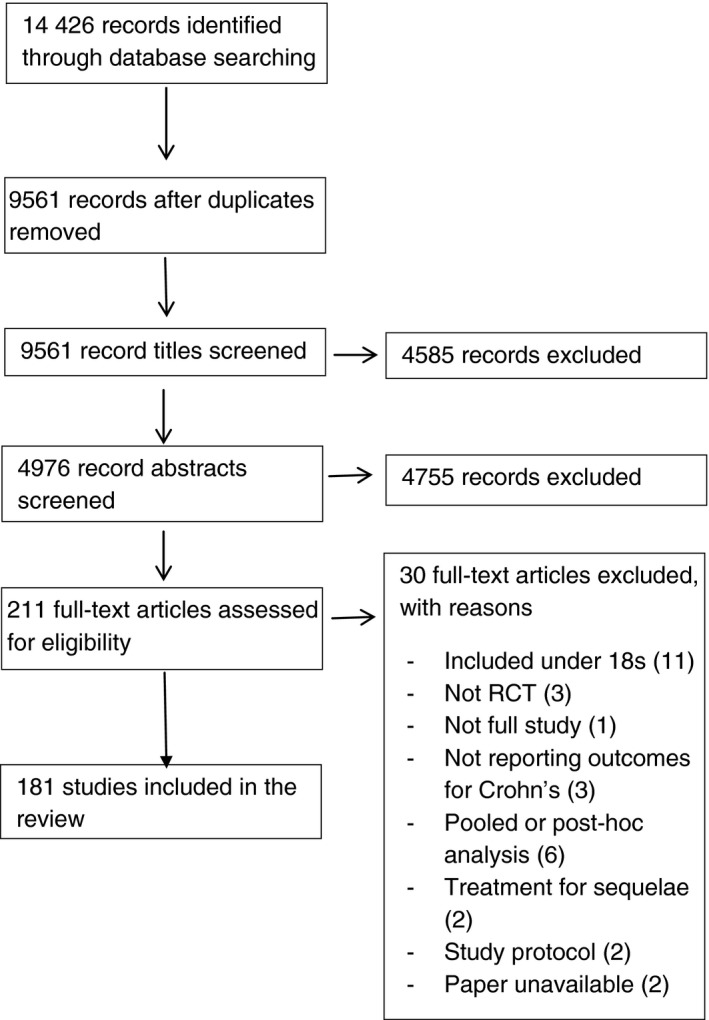
Preferred reporting items for systematic review and meta‐analyses (PRISMA) flow diagram

The search identified 9561 unique records (Figure 1) and included 181 RCTs (characteristics in Table S5). Induction of remission was the focus of 110 studies: 104 (94.5%) through medical^15^, ^16^, ^17^, ^18^, ^19^, ^20^, ^21^, ^22^, ^23^, ^24^, ^25^, ^26^, ^27^, ^28^, ^29^, ^30^, ^31^, ^32^, ^33^, ^34^, ^35^, ^36^, ^37^, ^38^, ^39^, ^40^, ^41^, ^42^, ^43^, ^44^, ^45^, ^46^, ^47^, ^48^, ^49^, ^50^, ^51^, ^52^, ^53^, ^54^, ^55^, ^56^, ^57^, ^58^, ^59^, ^60^, ^61^, ^62^, ^63^, ^64^, ^65^, ^66^, ^67^, ^68^, ^69^, ^70^, ^71^, ^72^, ^73^, ^74^, ^75^, ^76^, ^77^, ^78^, ^79^, ^80^, ^81^, ^82^, ^83^, ^84^, ^85^, ^86^, ^87^, ^88^, ^89^, ^90^, ^91^, ^92^, ^93^, ^94^, ^95^, ^96^, ^97^, ^98^, ^99^, ^100^, ^101^, ^102^, ^103^, ^104^, ^105^, ^106^, ^107^, ^108^, ^109^, ^110^, ^111^, ^112^, ^113^, ^114^, ^115^, ^116^, ^117^, ^118^ and six (5.5%) through surgical approaches^119^, ^120^, ^121^, ^122^, ^123^, ^124^(Table 1). Nine (of 110, 8.2%) induction studies solely treated patients with fistula with medical^36^, ^64^, ^79^, ^86^, ^91^, ^110^, ^113^ or surgical^119^, ^120^ therapies.

**Table 1:** Characteristics of randomised controlled trials in Crohn's disease

	Induction (n = 110)	Maintenance (n = 71)	Total (n = 181)
Trial participants	13 153	10 697	23 850
Trial year publication			
1979‐2008	78 (70.1)	47 (66.2)	125 (69.1)
2009‐2015	32 (29.1)	24 (33.8)	56 (30.9)
Country of lead author			
UK and Europe	61 (55.5)	40 (56.3)	101 (55.8)
USA and Canada	39 (35.5)	24 (33.8)	63 (34.8)
Rest of the world	10 (9.1)	7 (9.9)	17 (9.4)
Subgroup			
Medically induced	104 (94.5)	52 (73.2)	156 (86.2)
Fistula	7 (6.4)	1 (1.4)	
Surgically induced	6 (5.5)	19 (26.8)	25 (13.8)
Fistula	2 (1.8)	0	
Intervention of interest			
5‐ASAs	3 (2.7)	8 (11.3)	11 (6.1)
Antibiotics	8 (7.3)	3 (4.2)	11 (6.1)
Biologics	40 (36.4)	15 (21.1)	55 (30.4)
Corticosteroids	9 (8.2)	9 (12.7)	18 (9.9)
Immunosuppressants	7 (6.4)	7 (9.9)	14 (7.7)
Surgery	6 (5.5)	0	6 (3.3)
Dietary	16 (14.5)	5 (7.0)	21 (11.6)
CAM, prebiotics/probiotics	8 (7.3)	15 (21.1)	23 (12.7)
Combination interventions	6 (5.5)	8 (11.3)	14 (7.7)
Other	7 (6.4)	1 (1.4)	8 (4.4)
Comparator intervention			
Placebo	66 (60.0)	45 (63.4%)	111 (61.3)
Active	44 (40.0)	26 (36.6)	70 (38.7)
Follow‐up (wk)	16 (8.0‐25.1)	52.0 (48.0‐60.0)	25.1 (12.0‐52.0)

Maintenance of remission was the focus of 71 studies: 52 (73.2%) sought to maintain remission achieved through medical therapies^125^, ^126^, ^127^, ^128^, ^129^, ^130^, ^131^, ^132^, ^133^, ^134^, ^135^, ^136^, ^137^, ^138^, ^139^, ^140^, ^141^, ^142^, ^143^, ^144^, ^145^, ^146^, ^147^, ^148^, ^149^, ^150^, ^151^, ^152^, ^153^, ^154^, ^155^, ^156^, ^157^, ^158^, ^159^, ^160^, ^161^, ^162^, ^163^, ^164^, ^165^, ^166^, ^167^, ^168^, ^169^, ^170^, ^171^, ^172^, ^173^, ^174^, ^175^, ^176^ and 19 (26.8%) aimed to maintain surgically induced remission.^177^, ^178^, ^179^, ^180^, ^181^, ^182^, ^183^, ^184^, ^185^, ^186^, ^187^, ^188^, ^189^, ^190^, ^191^, ^192^, ^193^, ^194^, ^195^ One study aimed to maintain medically induced remission in fistula patients.^146^

In total, 23 850 patients were involved in the studies, with median follow‐up of 16 weeks (IQR: 8.0‐25.1) in induction studies and 52.0 weeks (IQR 48.0‐60.0) in maintenance studies. Over 30% of studies were published after 2009 (56 of 181, 30.9%). Biologics were the intervention of interest in 33.7% studies (61), either as monotherapy or in combination.

Table 2 shows a summary of the primary and secondary outcomes reported in Crohn's disease RCTs and highlights the wide range of outcomes and outcome measures. The reporting of outcomes not specified as primary or secondary endpoints was common (158 studies, 87.3%) and was consistent across the two time periods.

**Table 2:** Primary and secondary outcomes and measurement tools reported in randomised controlled trials in Crohn's disease

Outcome category	Primary or secondary outcomes	Measurement tools
Clinical or composite‐clinical	Clinical response (110) Clinical remission (85) Disease relapse or worsening (51) Fistula remission (10)/response (17) Corticosteroid‐sparing (14) Corticosteroid‐free remission (12)/response Recurrence (2) Sustained remission (11)/response (3) Combined clinical and endoscopic remission (1)/recurrence (3) Post‐operative recovery (2) Sustained corticosteroid‐free remission (2) Sustained fistula remission (2) Treatment compliance (2) Complete response (1)	Crohn's Disease Activity Index (141) Harvey Bradshaw Index (12) Physician Global Assessment (10) Perianal Disease Activity Index (6) Van Hees Activity Index (5) Severity and Activity Index (2) European Co‐operative Crohn's Disease Study based ranking system (1) Clinical recurrence grading scale (1) Dutch Index (1) International Organisation of Inflammatory Bowel Disease (IOIBD) score (1) Partial Harvey Bradshaw Index (1) Present Score (1)
Endoscopy	Endoscopic recurrence (21) Endoscopic response (16) Endoscopic mucosal healing (4) Endoscopic remission (1)	Rutgeerts endoscopic score (20) Crohn's Disease Endoscopic Index of Severity (12) Simple Endoscopic Score for Severity (4) D'Haen's endoscopic categories (1) Marteau endoscopic score (1)
Histology	Histologic recurrence (4) Tissue cytokine, leucocyte, receptor or gene expression (4) Histologic response (3) Histologic remission (1)	Average Histology Score (1) D'Haens‐Geboes score (4) Dieleman histological score (1) Histological Activity Score (1) Regueiro histology score (1)
Biomarkers	Serum C‐reactive protein (34) Serum erythrocyte sedimentation rate (16) Antidrug antibodies (10) Drug concentration and pharmacokinetics (8) Serum cortisol level (8) Serum full blood count and subsets (7) Serum protein concentrations (6) Intestinal permeability (4) Serum albumin (3) Autoantibodies (2) Faecal calprotectin (2) Serum lymphocyte count and subset expression (2) Serum cytokine or immunoglobulin levels (1)	
Economic outcomes	Cost of treatment (3) Utility (1)	Quality‐adjusted life years (1)
Patient‐reported outcomes	Quality of life (70) Pain (5) Defaecation functions (5) Bowel symptoms (2) Treatment compliance (2) Treatment acceptability (1)	IBDQ (55) SF‐36 (10) Patient Global Assessment (4) Visual analogue scale (4) Gastrointestinal Quality of Life Index (2) Hamilton Depression Scale (2) Short IBDQ (2) 16 PROM instruments were used once and are recorded in Table S9
Safety‐related outcomes	Adverse events (60) Abnormal laboratory or ECG parameters (25) Complications of surgery (2) Death (3)	Medical dictionary for regulatory activities^a^ Coding symbols for a thesaurus of adverse reactions terms^a^ WHO toxicity grading criteria^a^

AEs, adverse events; ECG, electrocardiogram; IBD, inflammatory bowel disease; IBDQ, inflammatory bowel disease questionnaire; IBS, irritable bowel syndrome; SF‐12, Short‐Form 12; SF‐36, Short‐Form 36; WHO, World Health Organisation.

a Number of reports not available.

### 3.2 Efficacy outcomes

#### 3.2.1 Clinical or composite‐clinical

**Figure 2:**
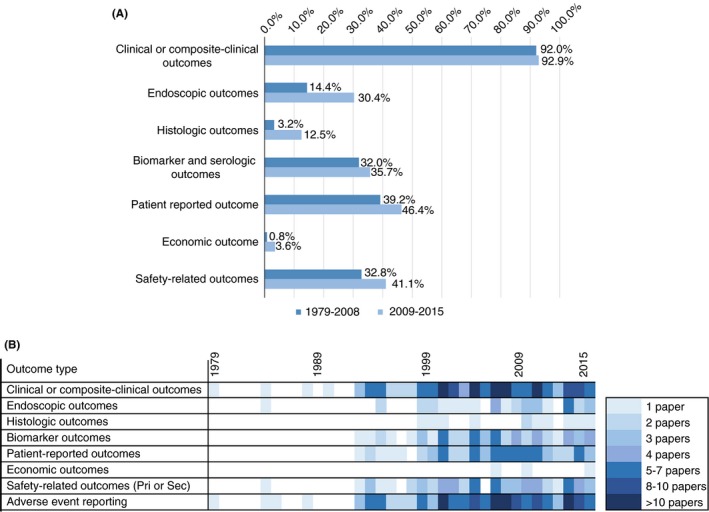
A, Proportion of Crohn's disease randomised controlled trials reporting key primary and secondary efficacy and safety outcomes, stratified by date of publication. B, Outcome reporting matrix for randomised controlled trials for Crohn's disease

Clinical or composite‐clinical efficacy outcomes were reported as primary or secondary endpoints in 92.3% of trials, which was consistent across the two time periods (Figure 2A).

Clinical response was reported by 70.0% (77) induction studies, 75 (of 104, 72.1%) medical and two (of six, 33.3%) surgical interventions (Table S6). Clinical response was reported less frequently in maintenance trials (31 of 71, 43.7%) but was more common in studies of maintenance of medically induced remission (26, 50%) than surgically induced (5, 19.3%). Clinical response was an outcome in 80% (eight) of studies of patients with fistulae.

Clinical remission was reported in 65.5% (72) of induction studies (all medical) and 19.7% (14) of maintenance studies. Clinical remission was not reported as a trial endpoint in surgical studies or studies of fistula patients.

Disease relapse or worsening was a primary or secondary outcome in 12.7% of induction studies (13 medical and one surgical) and 38 (73.1%) studies of maintenance of medically induced remission. Recurrence was reported in 14 (73.7%) maintenance studies of surgery‐induced remission and one (16.7%) surgical induction study.

Fistula response and remission were commonly reported in fistula studies (nine (90%) and six (60%), respectively). Overall, 14 (12.7%) induction studies and one (1.4%) maintenance study reported fistula response and 10 (9.1%) induction and two (2.8%) maintenance studies reported fistula remission.

Corticosteroid sparing and corticosteroid‐free remission were reported in 11 (10.6%) and eight (7.7%) medical induction studies and three (5.8%) and four (7.7%) maintenance studies of medically induced remission respectively. All studies, with one exception,^173^ were published prior to 2009.

The Crohn's Disease Activity Index (CDAI) dominated as the primary measurement tool for primary and secondary outcomes with 77.9% (141) of studies reporting its use, which was common across both induction (86, 78.2%) and maintenance (55, 77.5%) studies. The use of CDAI to measure primary and secondary outcomes reduced from 79.2% of studies pre‐2009, to 75.0% from 2009 onwards, although the chi‐squared value of 0.4 demonstrates that this was not a statistically significant result at the 95% confidence level. Outcome definitions using the CDAI were heterogeneous with 35 different definitions of response or remission reported (Table S6). CDAI 100 was the reported response measurement in 38 (21.0%) studies, only one before 2000.^21^ CDAI 70 was also reported in 38 (21.0%) studies, all but three after 2001. The remission benchmark CDAI <150, was the commonest (81, 44.8%), but reporting reduced between the two time periods (46.4%‐41.1%). Conversely, the reporting of CDAI 70 and CDAI 100 increased between the periods (20.8%‐21.4% and 16.8%‐30.4% respectively). The increase in CDAI 100 reporting was statistically significant at the 95% confidence level (chi‐squared value of 4.29). Fistula studies most commonly reported the change in CDAI score (5, 50%).

Other tools used less frequently to measure clinical response or remission include the Harvey Bradshaw Index,^19^, ^28^, ^48^, ^49^, ^87^, ^100^, ^118^, ^131^, ^157^, ^196^ Physician Global Assessments^19^, ^20^, ^25^, ^48^, ^99^, ^111^, ^180^ and the Van Hees Activity Index^19^, ^41^, ^66^, ^87^, ^131^ (Table 2). The Perianal disease Activity Index was used in four (40%) studies of fistula patients and in one nonfistula study. 36, 64, 91, 94, 120

There were 30 definitions of disease worsening or relapse, or recurrence using the CDAI, many of which required the CDAI to exceed a benchmark level such as 150, 200 or 250, with or without an increase from baseline score (Table S6). The need for additional therapy and/or surgery were commonly used to define worsening or relapse of disease.

Studies of penetrating disease most commonly used physician assessments of draining fistulas (50% [9, 90.0%] or 100% [6, 60%] reduction from baseline) as trial endpoints. Two (20.0%) studies of fistula patients used imaging techniques, MRI and diagnostic ultrasound, to assess response, one in each time period.^64^, ^120^

#### 3.2.2 Endoscopy

The reporting of endoscopic outcomes doubled between the two time periods, from 14.4% to 30.4% of studies (Figure 2A). This increase was statistically significant with a chi‐squared value of 6.31 (95% confidence level). Endoscopic outcomes were reported in 31% (22) of maintenance trials, with reporting more likely in studies of surgically (19, 100.0%) than medically (3, 5.8%) induced remission. Endoscopic outcomes were infrequently reported in induction trials (13, 11.8%) and in trials in penetrating disease (1, 10.0%).^120^ Reporting of endoscopic outcomes is a more recent phenomenon in induction trials, with their first use in a study reported in 2000, as compared with 1984 in maintenance trials.

Endoscopic recurrence was the most frequent endpoint, especially in maintenance trials (19, 26.8%). Only two induction studies^122^, ^123^ reported endoscopic recurrence, both of which involved surgery. Endoscopic response was more frequently reported in induction trials (10, 9.1%) than in maintenance trials (6, 8.5%). Endoscopic mucosal healing was reported in two (1.8%) induction^88^, ^111^ and two (2.8%) maintenance^162^, ^189^ studies and endoscopic remission in one (0.9%) induction study.^102^

Endoscopic recurrence was commonly defined with the Rutgeerts endoscopic score>=2 (14, 7.7%),^122^, ^179^, ^180^, ^181^, ^183^, ^185^, ^187^, ^188^, ^190^, ^191^, ^192^, ^193^, ^195^ although many benchmarks were used (Table S7). Endoscopic outcomes in induction (and fistula) trials report changes in the Crohn's Disease Endoscopic Index of Severity (CDEIS) score (9, 5.0%)^40^, ^69^, ^73^, ^76^, ^93^, ^98^, ^102^, ^112^, ^120^ or changes in the Simple Endoscopic Score for Crohn's Disease (SES‐CD) (4, 2.2%)^88^, ^98^, ^111^, ^120^ in place of the Rutgeerts score. The D'Haens^162^ and Marteau^191^ endoscopic scores were used infrequently.

Endoscopic outcomes were reported in 13.3% of studies (24) as additional outcomes and reporting increased pre‐2009 to 2009 onwards (12.0%‐16.1%), with the reporting growth exclusively in maintenance studies. This result was not statistically significant at the 95% confidence level (based on a chi‐squared test value of 1.58).

#### 3.2.3 Histology

Histology‐based outcomes have shown a statistically significant increase between the two periods (chi‐squared test statistic of 5.86) (Figure 2A), but remain uncommonly used (11, 6.1%) and are unused in studies of fistula patients. Three (medical) induction studies (1.7%)^62^, ^102^, ^112^ reported histologic response, one maintenance study (1.9%) of medically induced remission^162^ reported histologic remission and four maintenance studies (21.1%) of surgically induced remission^179^, ^182^, ^188^, ^193^ reported histologic recurrence. Three induction studies (1.7%)^40^, ^62^, ^97^ and one maintenance study (1.4%)^194^ reported outcomes related to cytokine expression in mucosal tissues. A number of histology scores are used including D'Haens,^62^, ^112^, ^182^, ^188^ Dieleman^102^ and Reguiero^193^ (Table S8). The reporting of histologic outcomes as additional outcomes increased between the time periods from 3.2% of studies to 7.1%, but this is not statistically significant at the 95% confidence level.

#### 3.2.4 Biomarkers

Biomarker outcomes were reported in 39 (35.5%) induction studies, 38 (36.5%) medical interventions and one (16.7%) surgical^120^ and 21 (29.6%) maintenance trials. Reporting has increased over time with 35.7% of trials since 2009 reporting a primary or secondary biomarker outcome (Figure 2A). However, this increase was not statistically significant. Only one (10.0%) study of penetrating disease reported a biomarker outcome.^120^ Serum C‐reactive protein was the most reported biomarker (34, 18.8%), followed by serum erythrocyte sedimentation (16, 8.8%). Faecal calprotectin was reported as an outcome in only two studies (1.1%),^66^, ^101^ one in each time period. The biomarker was an additional outcome in three (1.7%) further trials,^114^, ^115^, ^116^ all reported between 2014 and 2015.

#### 3.2.5 Patient‐reported outcomes

Patient‐reported outcomes (PROs) were reported in 47 (42.7%) induction studies, 45 (43.3%) medical induction studies and two (33.3%) surgical induction studies.^120^, ^121^ Reports of PROs were similar in studies of fistula patients (4, 40.0%). Primary or secondary PROs were reported in 28 (39.4%) maintenance studies, 24 (46.1%) of medically induced remission (46.1%), and four (21.1%) of surgically induced remission.^179^, ^189^, ^190^, ^194^ The use of PROs has increased over time, although not with statistical significance at the 95% confidence level, with almost half of RCTs reporting a primary or secondary PRO since 2009 (Figure 2A). Quality of life was the most common outcome, reported in 40.3% (73) of studies (Table S9). The Inflammatory Bowel Disease Questionnaire (IBD‐Q) was frequently used to record quality of life (59, 32.6%), and typically outcomes were specified as the final score or changes in the score (from baseline, mean or median). The use of IBD‐Q to measure PROs increased from 30.4% to 37.5% between 1979‐2008 and 2009‐2015. The growth in use was in maintenance studies (25.5%‐50.0%), whilst its use in induction studies reduced (33.3%‐28.1%). Whilst the overall change in IBDQ use and the decline in induction trials were not statistically significant at the 95% level, the increase in IBDQ studies in maintenance trials was significant (chi‐squared test value of 0.89, 0.28 and 4.25 respectively). Reporting of IBD‐Q in studies of fistula patients was in line with the overall average (3, 30.0%).

Other tools for measuring quality of life included the Short‐Form 36^40^, ^50^, ^106^, ^120^, ^121^, ^153^, ^160^, ^161^, ^169^, ^173^ and its components,^50^, ^121^, ^169^ Patient Global Assessments,^48^, ^91^, ^179^ the Gastrointestinal Quality of Life Index,^111^, ^121^ the Hamilton Depression Scale^81^, ^95^ and the Short IBDQ.^46^, ^120^ Patient diaries were used to measure outcomes related to bowel symptoms,^38^ defaecation functions^19^, ^46^, ^86^, ^96^ and pain^19^, ^46^, ^86^ (Table 2), with reports comparatively high (2,20%) in fistula patient studies.^86^, ^120^

### 3.3 Safety outcomes

Safety outcomes were specified as primary or secondary outcomes in 42 (38.2%) induction studies, 38 (36.5%) medical and four (66.7%) surgical.^119^, ^120^, ^121^, ^122^ Twenty‐two maintenance studies (31.0%) also reported primary or secondary safety outcomes. Safety outcome reporting increased from 32.8% of studies pre‐2009 to 41.1% between 2009 and 2015, although the increase was not statistically significant. Safety‐related primary and secondary outcomes were reported in three (30.0%) studies in fistula patients, all since 2010.^119^, ^120^, ^189^

Adverse events were the most common primary and secondary outcomes, reported in 39 (35.5%) induction and 22 (31%) maintenance studies. The reporting of adverse events as a primary or secondary endpoint was most frequently the totality of adverse events but some studies looked for specific treatment‐related adverse events or reported the stopping of treatment due to adverse events.

#### 3.3.1 Adverse events

Reporting of any adverse events occurred in 88 (80%) induction studies and 61 (85.9%) maintenance studies. All of the fistula studies reported adverse events. Reporting of adverse events increased slightly between the two periods from 80.0% to 87.5%. Serious adverse events were reported in 60 (54.5%) induction and 31 (43.7%) maintenance studies, and were higher in fistula patient trials (6, 60.0%). The reporting of serious adverse events in studies increased from 46.4% before 2009 to 58.9% from 2009 to 2015. Treatment‐related adverse events (including serious events), were reported in 69 (62.7%) induction and 44 (62%) maintenance studies. Six (60.0%) fistula studies reported treatment‐related adverse events. The reporting of treatment‐related adverse events (including serious) grew from 56.8% to 66.1% between the time periods respectively. None of the changes in reporting of adverse events was statistically significant at the 95% confidence level.

Gastrointestinal adverse events, including the exacerbation of Crohn's disease and gastrointestinal signs and symptoms, were the most commonly reported adverse events by MedDRA SOC, reported in 85 (77.3%) induction trials and 57 (80.3%) maintenance studies. The 10 most commonly reported adverse events by higher level group term (HLGT, a clinically relevant grouping) are shown in Table 3. Gastrointestinal signs and symptoms, including nausea, vomiting and pain, were reported as adverse events in 65.2% (118) of studies. Two other higher level group terms within the gastrointestinal conditions were in the 10 most reported: gastrointestinal inflammatory conditions (71, 39.2%), which includes Crohn's disease exacerbation as an adverse event, and gastrointestinal motility and defaecation conditions (63, 34.8%). Joint disorders, another higher level group term possibly related to Crohn's disease and the failure of treatment, were reported in 32.6% (59) studies.

**Table 3:** Ten most commonly reported MedDRA higher‐level group terms in randomised controlled trials in Crohn's disease, by intervention type

SOC	HLGT	All therapies rank	Medical induction rank	Surgical induction rank	Maintenance ‐ medical rank	Maintenance ‐ surgical rank	Fistula rank
Gastrointestinal disorders	Gastrointestinal signs and symptoms	1	1	4=	1	1	2=
Infections and infestations	Infections—pathogen unspecified	2	3	1	2	2=	1
Nervous system disorders	Headaches	3	2	4=	3	4=	4=
General disorders and administration conditions	General system disorders NEC	4	4		5		4=
Gastrointestinal disorders	Gastrointestinal inflammatory conditions	5	5	4=	6=	8=	2=
Gastrointestinal disorders	Gastrointestinal motility and defaecation conditions	6	7=	4=	4	2=	7=
Musculoskeletal and connective tissue disorders	Joint disorders	7	6		6=	4=	
General disorders and administration conditions	Fatal outcomes	8		4=^a^	8		
Nervous system disorders	Neurological disorders NEC	9=	10				7=
Skin and subcutaneous tissue disorders	Epidermal and dermal conditions	9=	7=				
Skin and subcutaneous tissue disorders	Skin appendage conditions			4=^a^	9	4=^b^	
General disorders and administration conditions	Body temperature conditions		7=	4=^a^			
Injury, poisoning and procedural complications	Procedural related injuries and complications NEC			2=			7=
Surgical and medical procedures	Therapeutic procedures and supportive care NEC			2=		8=	
Gastrointestinal disorders	Gastrointestinal stenosis and obstruction			4=^a^		8=	7=
Infections and infestations	Viral infectious disorders			4=^a^			7=
Musculoskeletal and connective tissue disorders	Musculoskeletal and connective tissue disorders NEC			4=^a^	10		4=

a Higher level group terms (HLGTs) reported in equal numbers only in surgical trials: anal and rectal conditions NEC, gastrointestinal haemorrhages NEC, gastrointestinal vascular conditions; protein and chemistry analyses NEC; appetite and general nutritional disorders; miscellaneous and site unspecified neoplasms malignant and unspecified), gastrointestinal therapeutic procedures; and embolism and thrombosis.

b HLGT reported in equal numbers but only in post‐operative maintenance trials: hepatobiliary investigations.

#### 3.3.2 Adverse events by intervention group

Five of the 10 most commonly reported adverse event groups for all therapies were also in the top 10 across all intervention groups (Table 3). Gastrointestinal signs and symptoms, infections (including anal abscess, post‐operative wound infection, urinary tract infection, upper respiratory tract infection and pneumonia) and headaches, the three most common adverse event groups for all trials, were ranked in the top four most reported for all trial subtypes. Gastrointestinal inflammatory conditions (Crohn's disease exacerbation) and gastrointestinal motility and defaecation were also commonly reported across all trial subtypes.

General system disorders, such as fatigue, pain, flushing, oedema, chills, influenza like illness, were commonly reported only in trials of medical induction or maintenance of medically induced remission interventions. Neurological disorders, such as dizziness, dysgeusia, paraesthesia, syncope and somnolence, and epidermal and dermal conditions, such as rash, pruritis, skin disorder, erythema and eczema, were in the list of ten most recorded adverse event groups across all trials, but were only commonly reported in medical induction trials.

Body temperature, specifically pyrexia, was one of the 10 most commonly reported adverse events in induction trials, but not maintenance. Procedural related injuries and complications, such as post‐operative ileus, post‐procedural haemorrhage, post‐procedural complication, infusion‐related reaction, anastomic leak and the need for therapeutic procedures and support care, such as surgery, hospitalisation and fistula repair, were only commonly reported in surgical induction and post‐operative maintenance trials.

#### 3.3.3 Adverse events by drug class

Gastrointestinal signs and symptoms, and infections were the only two adverse event groups that were consistently ranked in the 10 most commonly reported across all drug classes (including CAM, dietary and prebiotic/probiotic interventions) (Table 4). General system disorders, such as fatigue, asthenia, pain and chills, gastrointestinal inflammatory conditions (Crohn's disease exacerbation) and joint disorders were in the 10 most common adverse events across all but one drug class (corticosteroids, immunosuppressives and CAM respectively).

**Table 4:** Ten most commonly reported MedDRA higher level group terms in randomised controlled trials in Crohn's disease, by drug class

SOC	HLGT	All rank	5‐ASAs rank	Antibiotics rank	Biologics rank	Corticosteroids rank	Immunosuppressive rank	Dietary rank	CAM rank	Prebiotics/probiotics rank
Gastrointestinal disorders	Gastrointestinal signs and symptoms	1	1	1	1=	3	1=	2	2=	1
Infections and infestations	Infections—pathogen unspecified	2	10=	2	3	9=^a^	3	3=^a^	4=	3
Nervous system disorders	Headaches	3	4=		1=	5=^a^	1=	9=^a^	4=	
General disorders and administration conditions	General system disorders NEC	4	3	10=	5		4=	3=^a^	1	8=
Gastrointestinal disorders	Gastrointestinal inflammatory conditions	5	10=	6=	4	4		9=^a^	4=	4=
Gastrointestinal disorders	Gastrointestinal motility and defaecation conditions	6	2	6=	10=^a^			1	2=	2
Musculoskeletal and connective tissue disorders	Joint disorders	7	8=	6=	7=^a^	7=^a^	5=^a^	9=^a^		8=^a^
General disorders and administration conditions	Fatal outcomes	8	10=		6			9=^a^		4=
Nervous system disorders	Neurological disorders NEC	9=	4=	3=^a^			5=^a^	9=^a^	4=	
Skin and subcutaneous tissue disorders	Epidermal and dermal conditions	9=	4=	10=	10=^a^		5=^a^			
General disorders and administration conditions	Body temperature conditions			10=	7=^a^		5=^a^			
Endocrine disorders	Adrenal gland disorders					1				
Injury, poisoning and procedural complications	Injuries NEC								4=	4=
Surgical and medical procedures	Therapeutic procedures and supportive care NEC		10=	3=^a^				3=^a^		
Gastrointestinal disorders	Gastrointestinal stenosis and obstruction		8=					3=^a^		8=^a^
Musculoskeletal and connective tissue disorders	Musculoskeletal and connective tissue disorders NEC						5=	9=^a^	4=	
Skin and subcutaneous tissue disorders	Skin appendage conditions		4=			2				8=^a^
Surgical and medical procedures	Gastrointestinal therapeutic procedures							9=^a^	4=	4=
Infections and infestations	Fungal infectious disorders			6=						8=^a^
Investigations	Hepatobiliary investigations		10=				4=			8=^a^
Infections and infestations	Viral infectious disorders				10=^a^			9=^a^		
Musculoskeletal and connective tissue disorders	Muscle disorders		10=	10=						

a Higher level group terms (HLGTs) reported in equal numbers only in one drug class: 5‐ASAs trials: 10 = renal disorders (excl nephropathies); exocrine pancreas conditions. Antibiotic trials: 3 = bacterial infectious disorders. Biologics trials: 7 = toxicology and therapeutic drug monitoring; 10 = administration site reactions. Corticosteroids trials: 5 = lipid metabolism disorders. 7 = endocrine disorders of gonadal function. 9 = coagulopathies and bleeding diatheses (excl. thrombocytopenic); cornification and dystrophic skin disorders. Immunosuppressives trials: 5 = white blood cell disorders. Dietary trials: 3 = procedural related injuries and complications; gastrointestinal haemorrhages NEC. 9 = anaemias nonhaemolytic and marrow depression; lipid analyses; pregnancy, labour, delivery and postpartum conditions; suicidal and self‐injurious behaviours NEC; appetite and general nutritional disorders. Prebiotic/probiotic trials: 8 = cutaneous neoplasms benign; central nervous system vascular disorders; depressed mood disorders and disturbances; bronchial disorders (excl. neoplasms); peritoneal and retroperitoneal conditions; miscellaneous and site unspecified neoplasms malignant and unspecified.

Headaches were one of the 10 most common adverse event groups in all drug classes except antibiotics and prebiotics. Gastrointestinal motility and defaecation conditions were one of the 10 most commonly occurring adverse events across all drug groups, with the exception of corticosteroids and immunosuppressives.

Differences between drug classes and from the overall average were found. Skin appendage conditions were the fourth most common adverse events for 5‐aminosalycylic acid (5‐ASA) therapies, specifically alopecia and night sweats. Skin appendage conditions were the second most common adverse event grouping for corticosteroids, including acne, alopecia, hypertrichosis, hyperhidrosis and abnormal hair growth. Adrenal gland disorders, specifically Cushingoid, Cushing's syndrome, adrenal disorder and adrenal suppression, were the most common adverse events recorded by group and lipid metabolism disorders (lipohypertrophy) and were the fifth most common for corticosteroids. Neither adverse event group was commonly reported in any other drug class.

For the antibiotic drug class, bacterial infectious disorders (specifically clostridium difficile infection and furuncle) were the third most common adverse events, and were not common for any other drug class. Therapeutic procedures and supportive care, specifically surgery, hospitalisation and abscess drainage, were the third most commonly reported adverse event group for antibiotics, as it was for dietary treatments. Procedural related injuries and complications (procedural complication and feeding tube complication), gastrointestinal haemorrhages and gastrointestinal stenosis and obstruction, were also ranked the third most common adverse event groups for dietary treatments.

Commonly occurring adverse events, unique to immunosuppressive (ranked fifth and above) were white blood cell disorders, specifically leukopenia and lymphopenia. Body temperature conditions (pyrexia), musculoskeletal and connective tissue disorders (back pain, fistula and anal fistula) and hepatobiliary investigations (including alanine aminotransferase increased and liver function test abnormal) were also in the top five most commonly reported adverse events for immunosuppressives, although each was also commonly reported in other drug classes.

Injuries were the fourth most commonly reported adverse event groups for CAM and prebiotic or probiotic trials. However, with underlying terms including stab wound and road traffic accident, these are likely to be unrelated to the interventions.

Surgical interventions offer a different pattern of adverse events, as shown in Table 3. Infections are most commonly reported, followed by procedural related injuries and complications (including post‐operative ileus, post‐procedural haemorrhage, post‐procedural complication, infusion‐related reaction and anastomic leak), and therapeutic procedures and supportive care (Surgery, hospitalisation, adhesiolysis and abscess drainage). Gastrointestinal signs and symptoms, which are generally very commonly reported in drug classes (ranked first to third most common), are the fourth most common adverse event for surgical interventions, along with headaches and a number of other adverse event groups (Table 3).

#### 3.3.4 Study withdrawals

Withdrawals were most frequently reported due to adverse events (102, 56.4%) and least frequently for serious adverse events (7, 3.9%). Withdrawals due to treatment failure were reported in 41.4% of studies, and in 45.9% of studies for reasons related to noncompliance and loss to follow‐up, the reporting of both reduced between periods (45.6%‐32.1% and 52.0%‐32.1% respectively). Withdrawals due to treatment‐related adverse events (including serious) were reported by 13.8% (25) studies, but the proportion fell from 15.2% to 10.7% between the two time periods. The reduction in the reporting of study withdrawals was common across all categories except serious adverse events, which rose slightly from 3.2% to 5.4% of studies. It was not possible to test the increase in serious adverse events for statistical significance, as the requirement for 80% of numbers to be over five was not met. No changes in reporting reached statistical significance, except the reduction in withdrawals due to other reasons, which was significant at the 95% confidence level (chi‐squared test value of 6.14).

## 4 DISCUSSION

We conducted a comprehensive and independent systematic review of the outcomes and outcome measures reported in RCTs of interventions for Crohn's disease, summarising data from 181 RCTs. A key strength of our review was the focus on synthesising data on safety outcomes and adverse events, which goes beyond anything reported previously in the literature. Furthermore, we have not only described temporal trends in outcome reporting but have tested the statistical significance of these findings. Our results demonstrate that trialists have adopted a wide and variable approach to outcomes measurement and highlight commonalities and differences in the reporting of adverse events between a variety of interventions to induce or maintain remission in Crohn's disease. These results provide insights to guide future trial design and support core outcome set development.

The CDAI was developed over 40 years ago as a composite measure incorporating symptoms, signs and simple laboratory parameters.^197^ It was the dominant measurement instrument used in the published trials, but with substantial variation including 35 definitions of response or remission. Whilst this observation highlights a need for greater standardisation of endpoints, the CDAI per se is increasingly regarded as suboptimal as an endpoint for comparative effectiveness research and regulatory approval. The index does not correlate closely with objective signs of inflammation or with mucosal healing at endoscopy.^198^, ^199^ The time trends we observed in clinical trials outcomes reporting, specifically the statistically significant increase in endoscopy and histology outcomes reporting, illustrate how the emphasis is shifting towards inclusion of discrete, objective measures of the inflammatory process. Whilst the use of CDAI overall has shown nonstatistically significant growth, the use of CDAI 100 has significantly increased, highlighting a continued interest in this measure of response. This confirms that the CDAI 100, which is a 100 point reduction in CDAI score, is increasingly preferred to the CDAI 70 (70 point reduction in response), as a measure of response.^200^

C‐reactive protein is a routinely employed biomarker in clinical practice and was frequently reported among clinical trial outcomes, albeit rarely as a primary outcome (five studies). However, C‐reactive protein lacks sensitivity for active intestinal inflammation in Crohn's disease,^201^ and this limits its value as a primary endpoint. There remains active exploration of alternative serum markers of disease activity^202^ but our review suggests no strong candidate has emerged.

Stool biomarkers offer potential to reliably measure gut‐related inflammation and in recent years faecal calprotectin has become available in routine IBD practice.^203^ Uncertainty remains as to its performance properties particularly for measuring small bowel, rather than colonic, disease activity^204^ and research continues to explore other stool assays to measure the inflammatory process.^205^ Faecal calprotectin was reported as an endpoint in only two trials included in this review.^66^, ^101^

We found a statistically significant increase in the report of endoscopy and histology‐based outcome measures over time, albeit they remained at a low level and without emergence of a standardised approach. This heterogeneity likely reflects the current suboptimal psychometric properties of individual measurement tools, both for endoscopic and histologic scoring systems.^206^, ^207^ In addition to the cost and invasiveness of ileocolonoscopy, endoscopy is not able to fully characterise small bowel disease or quantify the overall extent of intestinal inflammation in Crohn's disease. There is a growing body of research on the potential use of quantitative imaging such as CT and MRI,^208^ but only one trial included in this review included radiological outcomes.^120^

Patient‐reported outcome measures (PROMs) were reported as endpoints in almost half of studies reported since 2009, although commonly as a secondary outcome (60, 33.1%) rather than a primary outcome (10, 5.5%). Questionnaires administered in clinical trials ranged from ‘generic” (eg EQ‐5D) and “disease specific” (eg IBD‐Q) health‐related quality of life instruments to tools focusing on individual domains (eg Fatigue Impact Score). The IBD‐Q was the most frequently reported PROM in the trials (85% of studies reporting PROMs) and there was a statistically significant increase in its use for measuring outcomes in maintenance studies over the time of the review (from 25.5% to 50.0%). However, it was not developed according to the latest FDA recommendations for product labelling claims.^209^ New disease‐specific PROMs tools are under development to meet the stringent guidelines and enable PROMs to support future regulatory approvals of licencing for Crohn's disease.

Our review covered data for safety outcomes in clinical trials and we found substantial heterogeneity in reporting, which highlights the challenges in categorising adverse events for a complex, chronic condition with a variable disease course and multisystem manifestations. Lack of treatment efficacy in Crohn's disease may manifest with a diversity of symptoms, which are difficult to distinguish from genuine treatment side effects. Many of the most commonly reported adverse events, such as gastrointestinal signs and symptoms and gastrointestinal inflammatory conditions may reflect disease course. Nevertheless, these data demonstrate differences in the adverse event profile of different intervention groups and should support renewed attempts to define disease‐ and intervention‐specific adverse events and to standardise safety outcomes as discrete endpoints. This is an important consideration for future core outcome set developers.

Our results highlight how the reporting of outcomes in trials in fistula patients align with overall reporting. The use of PROMS and safety‐related endpoints is common across all trials, regardless of disease type. Clinical response was less commonly measured by CDAI, and more frequently measured by fistula closure and the PDAI. These three outcome measures were the most commonly used in fistula trials identified by this review, which supports the findings of a recently developed core outcome set for fistulising disease.^8^ Biomarker, histology and endoscopy outcomes were rarely used in fistula trials and are not included in the core outcome set either, contrary to the general shift in outcomes reporting in Crohn's disease trials. However, patient reports (eg incontinence and drainage) were more common endpoints in trials of fistula patients than in nonfistula trials, and their importance is borne out in the core outcome sets, which lists several PROMs to be reported in future trials.

Our review independently supports the key findings of a recently published systematic review of outcomes in Crohn's disease.^9^ We confirm heterogeneity in definitions of response and remission and the need for a core outcome set to standardise endpoint definitions. Both studies identified the use of CDAI as the most popular outcome measurement tool overall and of IBD‐Q as the most commonly used PROM. Our results confirm statistically significant increases in the use of CDAI100 across all trials and of IBD‐Q reporting in maintenance trials across the time periods of the review. Similarly, the CDEIS and the SES‐CD are highlighted as endoscopic tools most used in induction trials and Rutgeerts in post‐surgical trials. Both reviews confirmed the common use of C‐reactive protein and increasing use of biomarkers.

However, our study had less restrictive inclusion criteria, leading to inclusion of a larger number of RCTs (181 vs 116) with a wider variety of interventions included. Our research included dietary, CAM, probiotic/prebiotic and surgical interventions, which results in extra heterogeneity in our findings. Our results are arguably more extensive, particularly in the reporting of safety‐related outcomes and adverse events, and go beyond the descriptive in the analysis of changes between time periods by including statistical testing. Furthermore, we focused on primary and secondary endpoints (with supplementary analysis of other outcomes), whereas Ma et al considered all outcomes in a singular analysis. This results in differences in the breadth and depth of scope of the reviews and some nuances in key findings between the two studies. For example, Ma et al found that a higher proportion of studies used CDAI, which likely reflects the requirement that trials must have used CDAI (or the Harvey‐Bradshaw Index) at enrolment to be included. Their focus on a more restricted range of therapies may also explain the higher proportion of studies reporting adverse events, as our results included trials of less traditional therapies. Ma et al also found that CDAI 100 was more prevalent as a measure of response than in our results (although we found a statistically significant increase in use over time), and reported an increased use of faecal calprotectin. These results may reflect some more recent trials included in their review. The use of CDAI as a requirement for trial inclusion in their systematic review reduces the ability of the Ma et al review to assess changes in the use of CDAI. We have been able to include such analysis in our paper, and confirm a statistically significant increase in CDAI100, whereas the use of CDAI overall has remained relatively consistent.

Our study has limitations. Whilst it includes a comprehensive listing of outcomes from available Crohn's disease trials, we cannot account for publication bias. The results would have been strengthened by the consideration of nonrandomised controlled trials and observational studies. In particular, this would help to characterise important longer term harms. We did not assess the validity or reliability of the outcome measures identified in the review, although this would form a part of any core outcome set development process.

Our study confirms the variability that exists in reporting of outcomes in published clinical trials of interventions for Crohn's disease. These data provide a comprehensive resource to support current efforts^7^ to redefine optimal outcomes and measurement tools to be included in future studies of comparative effectiveness.
