# Supplementary material for: Systematic review: outcomes and adverse events from randomised trials in Crohn's disease
Source: Aliment Pharmacol Ther. 2019 Mar 3;49(8):978–96. doi: 10.1111/apt.15174 (PMC6492112; doi:10.1111/apt.15174)
Supplement: Supplementary file 1 [file APT-49-978-s001.docx]

Supplementary Table 1: Cochrane Search Strategy for Crohn's Disease Outcomes in Adults (search completed 3rd November 2015)

| **#** | **Searches** | **Results** | **Search description** |
| --- | --- | --- | --- |
| **1** | MeSH descriptor: [Inflammatory Bowel Diseases] explode all trees | 2,092 | Inflammatory bowel disease |
| **2** | Inflammatory bowel disease* | 1,865 | Inflammatory bowel disease |
| **3** | #1 or #2 | 3,240 | Inflammatory bowel disease |
| **4** | MeSH descriptor: [Crohn disease] explode all trees | 1,034 | Crohn’s disease |
| **5** | Crohn* | 2,137 | Crohn’s disease |
| **6** | #4 or #5 | 2,137 | Crohn’s disease |
| **7** | #3 or #6 | 3,905 | Inflammatory bowel disease or Crohn’s disease |
| **8** | Outcome* | 241,924 | Outcomes |
| **9** | #7 and #8 | 1,794 | Inflammatory bowel disease or Crohn’s disease and outcomes |
| **10** | Not possible to limit on age |  |  |

Supplementary Table 2: Cumulative Index to Nursing and Allied Health Literature Search Strategy for Crohn's Disease Outcomes in Adults (search completed 3rd November 2015)

| **#** | **Searches** | **Results** | **Search description** |
| --- | --- | --- | --- |
| **1** | (MH “Inflammatory Bowel Diseases+”) | 8,376 | Inflammatory bowel disease |
| **2** | Inflammatory bowel disease* | 4,881 | Inflammatory bowel disease |
| **3** | S1 OR S2 | 9,322 | All inflammatory bowel disease |
| **4** | (MH “Crohn Disease”) | 4,017 | Crohn’s disease |
| **5** | Crohn* | 4,884 | Crohn’s disease |
| **6** | S4 or S5 | 4,884 | All Crohn’s disease |
| **7** | S3 or S6 | 9,769 | Inflammatory bowel disease or Crohn’s disease |
| **8** | Outcome* | 477,077 | Outcomes |
| **9** | S7 AND S8 | 1,310 | Inflammatory bowel disease or Crohn’s disease and outcomes |
| **10** | S9 with restriction for adults | 529 | Inflammatory bowel disease or Crohn’s disease and outcomes for adults |

Supplementary Table 3: EMBASE Search Strategy for Crohn's Disease Outcomes in Adults (search completed 3rd November 2015)

| **#** | **Searches** | **Results** | **Search description** |
| --- | --- | --- | --- |
| **1** | exp INFLAMMATORY BOWEL DISEASE/ | 98,256 | Inflammatory bowel disease |
| **2** | Inflammatory AND bowel AND disease.mp | 54,886 | Inflammatory bowel disease |
| **3** | 1 OR 2 | 119,712 | Inflammatory bowel disease |
| **4** | Exp CROHN DISEASE/ | 64,317 | Crohn’s disease |
| **5** | Crohn*.mp | 72,296 | Crohn’s disease |
| **6** | 4 OR 5 | 72,296 | Crohn’s disease |
| **7** | 3 OR 6 | 123,434 | Inflammatory bowel disease or Crohn’s disease |
| **8** | Outcome*.mp | 215,869 | Outcomes |
| **9** | 7 AND 8 | 16,198 | Inflammatory bowel disease or Crohn’s disease and outcomes |
| **10** | 9 [Limit to: (Human Age Groups Adult 18 to 64 years or Aged 64+ years) | 6,394 | Inflammatory bowel disease or Crohn’s disease and outcomes for adults |

Supplementary Table 4: MEDLINE Search Strategy for Crohn's Disease Outcomes in Adults (search completed 3^rd^ November 2015)

| **#** | **Searches** | **Results** | **Search description** |
| --- | --- | --- | --- |
| **1** | exp Inflammatory Bowel Diseases/ | 65,723 | Inflammatory bowel disease |
| **2** | Inflammatory bowel disease*.mp | 32,235 | Inflammatory bowel disease |
| **3** | 1 or 2 | 73,709 | Inflammatory bowel disease |
| **4** | exp Crohn disease/ | 33,044 | Crohn's disease |
| **5** | Crohn*.mp | 41,633 | Crohn's disease |
| **6** | 4 or 5 | 41,633 | Crohn's disease |
| **7** | 3 or 6 | 77,739 | Inflammatory bowel disease or Crohn’s disease |
| **8** | outcome*.mp | 1,539,674 | Outcomes |
| **9** | 7 and 8 | 9,153 | Inflammatory bowel disease or Crohn’s disease and outcomes |
| **10** | Limit 9 to “all adult (19 plus years)” | 5,710 | Inflammatory bowel disease or Crohn’s disease and outcomes for adults |

Supplementary Table 5: Characteristics of Randomised Controlled Trials in Crohn's Disease

| **Reference** | | **Country of study or lead author** | **Sample size** | **Disease behaviour** | **Follow up (weeks)** | **Intervention** |
| --- | --- | --- | --- | --- | --- | --- |
| **Induction (n=110)** | | | | | | |
| **Medical induction (n=104)** | | | | | | |
| Greenberg 1988 | | USA and Canada | 51 | CDAI >150, refractory | 52 | Parenteral versus defined formula nutrition |
| Wright 1990 | | USA and Canada | 11 | Patients requiring hospitalisation for acute exacerbation of Crohn's | 2 | Parenteral verus enteral nutrition |
| Rigaud 1991 | | UK and Europe | 30 | CDAI >150 | 52 | Enteral nutrition: elemental vs polymeric |
| Ewe 1993 | | UK and Europe | 42 | CDAI >150 | 16 | AZA and prednisolone |
| Singleton 1993 | | USA and Canada | 310 | CDAI 150-400 | 16 | Mesalamine |
| Wright 1993 | | Rest of world | 356 | Mild to moderate Crohn's, judged to need oral corticosteroids | 4 | Fluticasone propionate |
| Rutgeerts 1994 | | UK and Europe | 176 | CDAI >200 | 10 | Budesonide and prednisolone |
| Tremaine 1994 | | USA and Canada | 38 | CDAI 150-450 | 17 | Mesalamine |
| Royall 1994 | | USA and Canada | 40 | CDAI >250 | 52 | Amino acid based defined formula |
| Greenberg 1994 | | USA and Canada | 258 | CDAI >200 | 10 | Budesonide |
| Jewell 1994 | | UK and Europe | 147 | Chronically active Crohn's despite steroid treatment | 52 | Cyclosporine |
| Gross 1995 | | UK and Europe | 31 | CDAI 150-350 | 8 | 5-ASA |
| Mansfield 1995 | | UK and Europe | 44 | One symptom of active disease, CDAI >150 and one abnormal lab measurement | 4 | Amino acid versus oligopeptide diet |
| Middleton 1995 | | UK and Europe | 76 | HBI >=6 and elevated ESR, CRP and alpha-1-antichymotrypsin | 3 | Enteral feeds |
| Feagan 1995 | | USA and Canada | 141 | Chronically active Crohn's despite steroid treatment | 16 | Methotrexate |
| Targan 1997 | | USA and Canada | 108 | CDAI 220-400 | 12 | Infliximab |
| Frascio 1997 | | UK and Europe | 14 | CDAI >150 | 2 | Enteral and parenteral nutrition |
| Bar-Meir 1998 | | Rest of world | 201 | CDAI 150-350 | 8 | Budesonide and prednisolone |
| Colombel 1999 | | UK and Europe | 40 | CDAI 150-300 | 6 | Ciprofloxacin and mesalazine |
| Hond 1999 | | UK and Europe | 14 | Crohn's with increased intestinal permeability | 4 | Oral glutamine |
| D’Haens 1999 | | UK and Europe | 22 | CDAI 220-400, refractory | 4 | infliximab |
| **Present 1999 | | USA and Canada | 94 | Crohn's with single or multiple draining or abdominal fistulas of at least three months' duration | 34 | infliximab |
| Sandborn 1999 | | USA and Canada | 96 | CDAI 150-450, steroid treated | 18 | Azathioprine |
| Verma 2000 | | UK and Europe | 21 | CDAI >150, presence of bowel symptoms, at least one raised inflammatory marker and increased bowel activity on leukocyte bowel imaging | 4 | Elemental versus polymeric diet |
| Fedorak 2000 | | USA and Canada | 95 | CDAI 200-350 | 24 | rhuIL-10 |
| Schreiber 2000 | | UK and Europe | 329 | CDAI 200-400 | 8 | Tenovil |
| Leiper 2001 | | UK and Europe | 54 | CDAI >200 and serum C-reactive protein 10mg/l | 3 | Whole protein feed with long chain triglyceride content |
| Gordon 2001 | | UK and Europe | 30 | CDAI 150-450 | 12 | natalizumab |
| Schreiber 2001 | | UK and Europe | 78 | CDAI 200-400, steroid dependent | 26 | ISIS-2302 |
| Sandborn 2001 | | USA and Canada | 193 | CDAI 220-450 | 24 | CDP571 |
| Lomer 2001 | | UK and Europe | 20 | CDAI >150 | 17.3 | Low microparticle diet |
| Hawkes 2001 | | UK and Europe | 70 | CDAI 150-450 | 12 | Glyceryl trinitate (GTN) |
| Sandborn 2001 | | USA and Canada | 43 | CDAI 220-450 | 8 | Etanercept |
| Carty 2001 | | UK and Europe | 85 | CDAI 200-400 | 12 | Ridrogel |
| Goodgame 2001 | | USA and Canada | 31 | Crohn's disease | 52 | Ethambutol and clarithromycin |
| Tremaine 2002 | | USA and Canada | 200 | CDAI 200-450 | 10 | Budesonide |
| Steinhart 2002 | | USA and Canada | 134 | CDAI 200-400 | 8 | Ciprofloxacin and metronidazole |
| Yacyshyn 2002 | | USA and Canada | 22 | CDAI >220 | 52 | Alicaforsen |
| Hommes 2002 | | UK and Europe | 12 | CDAI 220-450 | 4 | CNI-1493, a guanylhydrazone |
| Arnold 2002 | | USA and Canada | 47 | CDAI >150 CDAI | 26 | Ciprofloxacin |
| Sakurai 2002 | | Rest of world | 36 | CDAI >150 | 6 | Enteral nutrition with low or high medium-chain triglycerides |
| Ardizzone 2003 | | UK and Europe | 54 | CDAI >200 | 26 | Methotrexate and azathioprine |
| Bamba 2003 | | Rest of world | 36 | International Organisation of Inflammatory Bowel Disease (IOIBD) rating >=2 and at least one abnormal inflammatory marker | 4 | Different fat-dose levels of enteral nutrition |
| Ghosh 2003 | | UK and Europe | 244 | CDAI 220-450 | 12 | natalizumab |
| Ito 2004 | | Rest of world | 36 | CDAI >150 and abnormal levels of serum C-reactive protein | 12 | anti-IL-6R mAb MRA |
| Joos 2004 | | UK and Europe | 51 | CDAI 150-350 | 12 | Traditional acupuncture |
| Herfarth 2004 | | UK and Europe | 104 | CDAI >150 | 6 | Budesonide |
| Mannon 2004 | | UK and Europe | 79 | CDAI 220-450 | 25 | anti-interleukin-12 |
| Sandborn 2004 | | USA and Canada | 396 | CDAI 220-450 | 28 | CDP571 |
| **West 2004 | | UK and Europe | 24 | Crohn's disease complicated by single or multiple draining perianal fistulae | 18 | Ciprofloxacin and infliximab |
| Winter 2004 | | USA and Canada | 92 | CDAI 220-450 | 12 | CDP870 |
| Schreiber 2005 | | UK and Europe | 291 | CDAI 220-450 | 20 | certolizumab pegol |
| Lomer 2005 | | UK and Europe | 83 | CDAI >150 | 52 | Low microparticle diet |
| Korzenik 2005 | | USA and Canada | 124 | CDAI 220-475 | 34.3 | Granulocyte-macrophage colony-stimulating factor (GM-CSF) |
| Reinsich 2006 | | UK and Europe | 45 | CDAI 250-450 | 25.1 | fontolizumab |
| Margalit 2006 | | USA and Canada | 31 | CDAI 220-400 | 27 | Autologous colonic proteins |
| Prantera 2006 | | UK and Europe | 83 | CDAI 220-400 | 16 | Rifaximin |
| Schroder 2006 | | UK and Europe | 19 | Refractory to or dependent on corticosteroids | 48 | infliximab and methotrexate |
| Hommes 2006 | | UK and Europe | 133 | CDAI 250-450 | 26 | fontolizumab |
| Hanauer 2006 | | USA and Canada | 299 | CDAI 220-450 | 4 | Adalimumab |
| Lemann 2006 | | UK and Europe | 115 | CDAI >150, steroid dependent | 52 | infliximab |
| Rutgeerts 2006 | | UK and Europe | 207 | CDAI 250-400 | 20 | onercept |
| Schreiber 2006 | | USA and Canada | 284 | CDAI 220-450 | 8 | BIRB 796 |
| Herrlinger 2006 | | UK and Europe | 52 | CDAI >220 | 12 | rhIL-11 and prednisolone |
| Reinshagen 2007 | | UK and Europe | 58 | CDAI 150-450 | 24 | Azathioprine and azathioprine dose adaption |
| Mansfield 2007 | | UK and Europe | 84 | CDAI 200-400 | 12 | Lenalidomide |
| Sandborn 2007 | | USA and Canada | 325 | CDAI 220-450 | 4 | Adalimumab |
| Hafer 2007 | | UK and Europe | 31 | Active Crohn's | 17.3 | lactulose syrup |
| Targan 2007 | | USA and Canada | 509 | CDAI 220-450 and elevated CRP | 12 | natalizumab |
| Omer 2007 | | UK and Europe | 40 | CDAI >170 | 20 | Wormwood |
| **Hart 2007 | | UK and Europe | 19 | Patients with single or multiple draining perianal fistulas or perianal or anal ulcerating disease without fistulas | 24 | Tacrolimus |
| Sandborn 2008 | | USA and Canada | 104 | CDAI 220-450 | 28 | ustekinumab |
| Leiper 2008 | | UK and Europe | 41 | CDAI >200 | 12 | Clarithromycin |
| D’Haens 2008 | | UK and Europe | 133 | CDAI >200 | 104 | Immunosuppressives and Infliximab |
| **Fukuda 2008 | | Rest of world | 62 | At least one intractable active anal fistula | 8 | Spherical carbon adsorbant |
| Feagan 2008 | | USA and Canada | 185 | CDAI 220-400 | 25.7 | vedolizumab |
| **Thia 2009 | | USA and Canada | 27 | Perianal Crohn's with 1 or more open actively draining perianal fistula | 10 | Ciprofloxacin and metronidazole |
| Dotan 2010 | | Rest of world | 152 | CDAI 250-400 | 8.1 | Semapimod |
| Steed 2010 | | UK and Europe | 35 | CDAI 150-450 | 26 | Synbiotic B.longum and Synergy 1 |
| Van der Woude 2010 | | UK and Europe | 40 | CDAI 220-450, elevated CRP and endoscopic confirmation | 27.9 | NI-0401 |
| Maeda 2010 | | UK and Europe | 74 | PCDAI score of >=5 | 4 | Metronidazole |
| Sands 2010 | | USA and Canada | 220 | CDAI 220-450 | 30.1 | Apilimod mesylate |
| Buchman 2010 | | USA and Canada | 100 | CDAI 220-450 | 24 | Teduglutide |
| Krebs 2010 | | UK and Europe | 20 | CDAI >200 | 6 | Wormwood |
| Tromm 2011 | | UK and Europe | 311 | CDAI 200-400 | 8 | Budesonide and mesalamine |
| Sandborn 2011 | | USA and Canada | 439 | CDAI 220-450 | 6 | certolizumab pegol |
| Benjamin 2011 | | UK and Europe | 103 | CDAI >220 and an additional marker of inflammation | 4 | Prebiotic fructo-oligosaccharides (FOS) |
| Smith 2011 | | USA and Canada | 34 | CDAI >220 | 24 | naltrexone |
| Prantera 2012 | | UK and Europe | 402 | CDAI 220-400 | 24 | Rifaximin |
| Hueber 2012 | | UK and Europe | 59 | CDAI 220-450 | 16 | Secukinumab |
| Sands 2013 | | USA and Canada | 235 | CDAI 220-450 | 24 | Granulocyte / monocyte apheresis |
| Naftali 2013 | | Rest of world | 21 | CDAI 200-450 | 10 | Cannabis |
| Suzuki 2013 | | Rest of world | 77 | CDAI >200 | 10 | Budesonide |
| Sandborn 2013 | | USA and Canada | 36 | Confirmed diagnosis of Crohn's | 26 | Trichuris suis ova |
| Brotherton 2014 | | USA and Canada | 7 | partial Harvey Bradshaw Index >=3 | 4 | Whole wheat fibre diet |
| Sandborn 2014 | | USA and Canada | 139 | CDAI 220-450 | 8 | Tofacitinib |
| **Dewint 2014 | | UK and Europe | 76 | Active perianal fistulising Crohn's | 24 | Adalimumab and ciprofloxacin |
| **Reinisch 2014 | | UK and Europe | 249 | At least one draining perianal fistula, CDAI <400 | 24 | Spherical carbon adsorbant |
| Sands 2014 | | USA and Canada | 416 | CDAI 220-400 and one of the following: elevated C-reactive protein, endoscopy documented ulcerations or elevated faecal calprotectin and features of clinical activity | 22 | vedolizumab |
| Dignass 2014 | | UK and Europe | 471 | CDAI 200-400 | 10 | Budesonide |
| Bao 2014 | | Rest of world | 92 | CDAI 150-350 | 24 | Acupuncture and moxibustion |
| D’Haens 2015 | | UK and Europe | 180 | CDAI 220-450 | 12 | Laquinimod |
| Monteleone 2015 | | UK and Europe | 166 | CDAI 220-400 | 12 | Mongerson |
| Vande Casteele 2015 | | UK and Europe | 251 | treated with infliximab for at least 14 weeks and in a stable clinical response | 52 | infliximab |
| **Surgical induction (n=6)** | | | | | | |
| Maartense 2006 | | UK and Europe | 60 | Patients undergoing elective ilocolonic resection | 13 | Laparascopic versus open resection |
| East 2007 | | UK and Europe | 13 | Symptomatic strictures suitable for colonscopic dilation | 52 | Intrastricture steroid after balloon dilatation of strictures |
| Mcleod 2009 | | USA and Canada | 170 | Patients undergoing elective ilocolonic resection | 52 | End to end and side to side anastomosis |
| **Grimaud 2010 | | UK and Europe | 77 | CDAI <250 and at least one draining perianal fistula | 16 | Fibrin glue |
| Zurbuchen 2013 | | UK and Europe | 67 | Crohn's patients with ileitis terminalis who underwent elective ileocecal resection | 0 | End to end and side to side anastomosis |
| **Molendijk 2015 | | UK and Europe | 21 | Actively draining fistulising Crohn's with 1-2 internal openings and 1-3 fistula tracts and CDAI <250 | 24 | Mesenchymal stromal cells (MSC) |
| **Maintenance (n=71)** | | | | | | |
| **Maintenance studies of medically induced maintenance (n=52)** | | | | | | |
| Singleton 1979 | | USA and Canada | 89 | CDAI >150 | 26 | Prednisone and sulfasalazine |
| Malchow 1984 | | UK and Europe | 452 | Active Crohn's or quiescent | 104 | Sulfasalazine and / or methylprednisolone |
| Levenstein 1985 | | UK and Europe | 58 | Non-stenoising Crohn's | 104 | Low residue or normal diet |
| Bresci 1994 | | UK and Europe | 66 | CDAI <150 | 208 | 5-ASA |
| Feagan 1994 | | USA and Canada | 305 | Active crohn's with symptoms requiring treatment with steroids or 5-ASA | 78 | Cyclosporine |
| Schreiber 1994 | | UK and Europe | 60 | CDAI <150 for at least two months | 52 | 4-ASA and 5-ASA |
| Stange 1995 | | UK and Europe | 182 | Stratified: CDAI <200 and CDAI >200 | 65 | Cyclosporine |
| Belluzzi 1996 | | UK and Europe | 78 | CDAI <150 for 3 months - 2 years plus elevated inflammation marker | 52 | Fish oil |
| Greenberg 1996 | | USA and Canada | 105 | CDAI <150 | 52 | Budesonide |
| Sutherland 1997 | | USA and Canada | 293 | CDAI <150 with no symptoms for the past 30 days | 48 | Mesalamine |
| Ferguson 1998 | | UK and Europe | 75 | CDAI <150 | 52 | Budesonide |
| Arora 1999 | | USA and Canada | 33 | Steroid dependent for at least 6 months | 52 | Methotrexate |
| Guslandi 2000 | | UK and Europe | 32 | CDAI <150 for at least three months | 26 | Probiotic Saccharomyces boulardii |
| Green 2001 | | UK and Europe | 141 | In remission for at least 1 month (exhibiting no or mild symptoms) | 52 | Flexible dose budesonide |
| Mahmud 2001 | | UK and Europe | 328 | CDAI <150 for at least one month | 52 | Olsalazine |
| Cortot 2001 | | UK and Europe | 120 | CDAI <200 | 22 | Budesonide |
| Hanauer 2002 | | USA and Canada | 335 | CDAI 220-400 | 54 | infliximab |
| Mantzaris 2003 | | UK and Europe | 57 | CDAI <150, steroid dependent | 52 | Budesonide and mesalamine |
| Keller 2004 | | UK and Europe | 108 | At least one active episode of disease in the last 2 years | 104 | Psychotherapy and relaxation therapy |
| **Sands 2004 | | USA and Canada | 282 | Crohn's with single or multiple draining or abdominal fistulas of at least three months' duration | 54 | infliximab |
| Schultz 2004 | | USA and Canada | 11 | CDAI 150-300 | 26 | Probiotic Lactobacillus GG |
| Vilien 2004 | | UK and Europe | 29 | Crohn's in remission and on AZA for at least 2 years | 52 | Azathioprine |
| Lemann 2005 | | UK and Europe | 83 | In clinical remission induced by azathioprine for >=42 months | 78 | Azathioprine |
| Feagan 2005 | | USA and Canada | 71 | CDAI <150 and receiving corticosteroid therapy | 16 | CDP571 |
| Hanauer 2005 | | USA and Canada | 110 | CDAI <150 | 52 | Budesonide |
| Sandborn 2005 | | USA and Canada | 905 | CDAI 220-450 | 60 | natalizumab |
| Feagan 2006 | | USA and Canada | 271 | CDAI <150, steroid dependent | 34 | CDP571 |
| Takagi 2006 | | Rest of world | 51 | CDAI <150 | 104 | Half elemental diet |
| Colombel 2007 | | UK and Europe | 778 | CDAI 220-450 | 60 | Adalimumab |
| Sandborn 2007 | | USA and Canada | 55 | CDAI <150 | 52 | Adalimumab |
| Ng 2007 | | USA and Canada | 32 | Mildly active or disease in remission | 13 | Exercise: walking |
| Schreiber 2007 | | UK and Europe | 425 | CDAI 220-450 | 26 | certolizumab pegol |
| De Jong 2007 | | UK and Europe | 157 | CDAI <150 for 3-18 months | 52 | Budesonide |
| Selby 2007 | | Rest of world | 213 | CDAI >200 | 52 | Clarithromycin, Rifabutin and Clofazimine |
| Garcia 2008 | | Rest of world | 34 | CDAI <150 | 13 | Probiotic Saccharomyces boulardii |
| Feagan 2008 | | USA and Canada | 363 | CDAI <150 | 58 | Omega 3 free fatty acids |
| Mantzaris 2009 | | UK and Europe | 77 | CDAI <150, steroid dependent | 52 | Azathioprine and budesonide |
| Rossi 2009 | | UK and Europe | 67 | CDAI <150 | 56 | IFN beta-la |
| Takagi 2009 | | Rest of world | 51 | CDAI <150 | 104 | Half elemental diet |
| Valentine 2009 | | USA and Canada | 156 | CDAI 150-450 | 26 | Granulocyte-macrophage colony-stimulating factor (GM-CSF) |
| Sandborn 2010 | | USA and Canada | 329 | CDAI 220-450 | 26 | certolizumab pegol |
| Jorgensen 2010 | | UK and Europe | 94 | CDAI <150 and biochemical signs of quiescent CD | 52 | Vitamin D3 |
| Prantera 2011 | | UK and Europe | 73 | CDAI <150 | 24 | Beclomethasone dipropionate (BDP) |
| Holtmeier 2011 | | UK and Europe | 82 | CDAI <150 | 64 | Boswellia serrata |
| Watanabe 2012 | | Rest of world | 90 | CDAI 220-450 | 52 | Adalimumab |
| Keefer 2012 | | USA and Canada | 28 | CDAI <150 | 6 | Project management |
| Keshav 2013 | | UK and Europe | 436 | CDAI 250-450 and elevated C-reactive protein | 52 | Vercinon |
| Bourreile 2013 | | UK and Europe | 165 | CDAI <150 after induction therapy | 65 | Probiotic Saccharomyces boulardii |
| Jigaranu 2014 | | UK and Europe | 168 | CDAI 220-400 | 48 | Rifaximin |
| Feagan 2014 | | USA and Canada | 126 | Active Crohn's being treated with prednisone | 50 | Methotrexate and infliximab |
| Piche 2014 | | UK and Europe | 37 | Normal global assessment by a clinician, normal C-reactive protein, erythrocyte sedimentation rate, platelet count and white cell count, no use of corticiosteroids in the past 12 months, CDAI <150 and normal mucosa | 8.6 | Osteopathy |
| Wenzl 2015 | | UK and Europe | 52 | CDAI <150 and azathioprine therapy >= 4 years | 104 | Azathioprine |
| **Maintenance studies of surgically induced remission (n=19)** | | | | | | |
| Brignola 1995 | | UK and Europe | 87 | Post curative resection | 52 | Mesalamine |
| McLeod 1995 | | USA and Canada | 163 | Post surgical resection | 312 | Mesalamine |
| Ewe 1999 | | UK and Europe | 83 | Post curative resection for ileal, ileo-colonic or colonic Crohn's | 52 | budesonide |
| Hellers 1999 | | UK and Europe | 129 | Patients scheduled for resectional surgery for ileocolonic Crohn's | 52 | Budesonide |
| Lochs 2000 | | UK and Europe | 324 | Post surgical resection | 78 | Mesalamine |
| Colombel 2001 | | UK and Europe | 65 | First resectional surgery for ileal or ileocolonic Crohn's | 16 | Tenovil |
| Prantera 2002 | | UK and Europe | 55 | Undergone recent curative resection | 52 | Probiotic Lactobacillus rhamnosus GG |
| Caprilli 2003 | | UK and Europe | 206 | Post surgical resection | 52 | Mesalazine |
| Hanauer 2004 | | USA and Canada | 131 | Patients schedules for resection | 104 | 6-mercaptopurine or mesalamine |
| Marteau 2006 | | UK and Europe | 98 | Undergone recent curative resection | 26 | Probiotic Lactobacillus johnsonii LA1 |
| D’Haens 2008 | | UK and Europe | 81 | Post ileal or ileocolonic resection with ileocolonic anastomosis | 52 | Metronidazole and azathioprine |
| Regueiro 2009 | | USA and Canada | 24 | Patients with ileal or ileocolonic Crohn's undergoing resection | 60 | infliximab |
| Reinisch 2010 | | UK and Europe | 78 | CDAI <200 and endoscopic recurrence Rutgeerts grade >=2 | 52 | Azathioprine |
| Savarino 2013 | | UK and Europe | 51 | Undergoing resection | 104 | Adalimumab |
| Herfarth 2013 | | USA and Canada | 33 | Ileal or ileocolonic resection with ileocolonic anastomosis | 26 | Ciprofloxacin |
| Ren 2013 | | Rest of world | 39 | CDAI <150 since resection | 52 | Tripterygium wilfordii polyglycoside |
| Armuzzi 2013 | | UK and Europe | 22 | Post curative resection | 52 | Azathioprine and infliximab |
| Fedorak 2015 | | USA and Canada | 120 | Post resection with margins macroscopically free of disease | 52 | Probiotic VSL#3 |
| Zhu 2015 | | Rest of world | 90 | Crohn's undergoing macroscopic disease resection | 52 | Tripterygium wilfordii Hook f. |
| Note: CDAI, Crohn’s disease activity index; IOIBD, International Organisation of Inflammatory Bowel Disease; HBI, Harvey Bradshaw Index; ESR, erythrocyte sedimentation rate; CRP, C-reactive protein; PDAI, perianal disease activity index; **, study involved only patients with fistula.  ** Trials in fistula patients | | | | | | |

Supplementary Table 6: Primary and Secondary Clinical and Composite-Clinical Efficacy Outcomes in Crohn's Disease Randomised Controlled Trials

| **Reference** | | **Outcome** | | **Outcome measurement** | | **Measurement tool** | |
| --- | --- | --- | --- | --- | --- | --- | --- |
| **Induction (n=101)** | | | | | | | |
| **Medical induction (n=95)** | |  | |  | |  | |
| Greenberg 1988 | | Disease relapse or worsening | | Need for additional therapy or surgery | |  | |
|  | | Disease relapse or worsening | | CDAI >250 | | CDAI | |
| Wright 1990 | | Remission | | CDAI <150 | | CDAI | |
| Ewe 1993 | | Response | | Change in CDAI score | | CDAI | |
|  | | Response | | Change in Dutch index score | | Dutch index | |
|  | | Response | | Change in SAI score | | SAI | |
|  | | Corticosteroid sparing | | Steroid dose | |  | |
|  | | Remission | | CDAI <150 | | CDAI | |
| Singleton 1993 | | Response | | CDAI decrease by >=50 points | | CDAI | |
|  | | Response | | Mean CDAI score | | CDAI | |
|  | | Response | | Mean HBI score | | HBI | |
|  | | Response | | Mean PGA of degree of illness on a Visual Analogue Scale | | PGA | |
|  | | Response | | Mean VHAI score | | VHAI | |
|  | | Remission | | CDAI <150 and a reduction of >=50 | | CDAI | |
| Wright 1993 | | Remission | | PGA of disease severity | | PGA | |
| Rutgeerts 1994 | | Remission | | CDAI <150 | | CDAI | |
|  | | Response | | CDAI <150 or CDAI 100 | | CDAI | |
| Tremaine 1994 | | Response | | CDAI <150 or CDAI 70 | | CDAI | |
|  | | Disease relapse or worsening | | CDAI increase of >=100 from baseline | | CDAI | |
|  | | Remission | | CDAI <150 and CDAI 70 | | CDAI | |
| Greenberg 1994 | | Remission | | CDAI <150 | | CDAI | |
| Jewell 1994 | | Corticosteroid-free response | | Clinician grades response as clinically significant improvement and steroid withdrawal | | PGA | |
|  | | Disease relapse or worsening | | Development of new fistula or abscess | |  | |
|  | | Disease relapse or worsening | | Need for additional therapy or surgery | |  | |
|  | | Remission | | Clinician grades response as freedom from clinical symptoms | | PGA | |
| Gross 1995 | | Remission | | CDAI <150 and a reduction of >=60 | | CDAI | |
| Middleton 1995 | | Remission | | HBI <=3 | | HBI | |
| Feagan 1995 | | Response | | Mean CDAI score | | CDAI | |
|  | | Corticosteroid sparing | | Mean daily prednisone dose | |  | |
|  | | Corticosteroid-free remission | | CDAI <150 and withdrawal of corticosteroids | | CDAI | |
| Targan 1997 | | Response | | CDAI 70 | | CDAI | |
| Bar-Meir 1998 | | Response | | CDAI <150 or decrease of >=60 with no steroid side effects | | CDAI | |
|  | | Response | | CDAI <150 or decrease of >=60 with steroid side effects | | CDAI | |
| Colombel 1999 | | Remission | | CDAI <150 and a reduction of >75 | | CDAI | |
| Present 1999** | | Response | | Change in CDAI score | | CDAI | |
|  | | Response | | Change in PDAI score | | PDAI | |
|  | | Fistula remission | | Closure of all active draining anal fistulas at baseline | |  | |
|  | | Fistula response | | Reduction of 50% in draining fistula | |  | |
| Sandborn 1999 | | Response | | CDAI 70 | | CDAI | |
|  | | Corticosteroid sparing | | Mean daily prednisone dose | |  | |
|  | | Corticosteroid sparing | | Withdrawal of steroids | |  | |
|  | | Corticosteroid-free remission | | CDAI <150 and withdrawal of corticosteroids | | CDAI | |
|  | | Response | | Mean CDAI score | | CDAI | |
|  | | Remission | | CDAI <150 | | CDAI | |
| Verma 2000 | | Remission | | CDAI <150 or CDAI 100, no bowel symptoms and normal CRP | | CDAI | |
| Fedorak 2000 | | Combined clinical and endoscopic remission | | CDAI <150 and improvement or resolution in endoscopic appearance | | CDAI and PGA | |
| Schreiber 2000 | | Response | | CDAI 100 | | CDAI | |
|  | | Remission | | CDAI <150 and CDAI 100 | | CDAI | |
| Leiper 2001 | | Remission | | CDAI <150 | | CDAI | |
|  | | Response | | CDAI 70 | | CDAI | |
|  | | Response | | HBI <=3 | | HBI | |
|  | | Response | | Change in VHAI score | | VHAI | |
| Gordon 2001 | | Remission | | CDAI <150 | | CDAI | |
|  | | Disease relapse or worsening | | Need for additional therapy | |  | |
|  | | Response | | Change in CDAI score | | CDAI | |
| Schreiber 2001 | | Remission | | CDAI <150 and low dose corticosteroids | | CDAI | |
|  | | Corticosteroid sparing | | Changes from baseline in daily steroids consumed | |  | |
|  | | Corticosteroid-free remission | | CDAI <150 and withdrawal of corticosteroids | | CDAI | |
|  | | Response | | CDAI 100 | | CDAI | |
|  | | Response | | CDAI 70 | | CDAI | |
| Sandborn 2001(1) | | Fistula response | | Reduction of 50% in draining fistula | |  | |
|  | | Response | | CDAI 100 | | CDAI | |
|  | | Response | | CDAI 70 | | CDAI | |
|  | | Remission | | CDAI <150 | | CDAI | |
| Lomer 2001 | | Corticosteroid sparing | | Median corticosteroid usage | |  | |
|  | | Response | | Mean CDAI score | | CDAI | |
| Hawkes 2001 | | Response | | Change in CDAI score | | CDAI | |
| Sandborn 2001(2) | | Fistula remission | | Closure of all draining fistulas | |  | |
|  | | Fistula response | | Reduction of 50% in draining fistula | |  | |
|  | | Response | | CDAI <150 or CDAI 70 | | CDAI | |
|  | | Response | | CDAI score | | CDAI | |
|  | | Remission | | CDAI <150 | | CDAI | |
| Carty 2001 | | Response | | Mean CDAI score | | CDAI | |
|  | | Response | | Mean HBI score | | HBI | |
|  | | Response | | PGA of change in clinical condition | | PGA | |
|  | | Remission | | CDAI <150 | | CDAI | |
| Goodgame 2001 | | Disease relapse or worsening | | Need for hospitalisation due to worsening Crohn's | |  | |
|  | | Disease relapse or worsening | | Need for surgery | |  | |
|  | | Response | | Change in HBI | | HBI | |
| Tremaine 2002 | | Response | | CDAI 100 | | CDAI | |
|  | | Remission | | CDAI <150 | | CDAI | |
|  | | Response | | Change in CDAI score | | CDAI | |
| Steinhart 2002 | | Response | | Change in CDAI score | | CDAI | |
|  | | Remission | | CDAI <150 | | CDAI | |
| Yacyshyn 2002 | | Corticosteroid sparing | | Corticosteroid use over time | |  | |
|  | | Corticosteroid-free remission | | CDAI <150 and no need for corticosteroids or immunosuppressives | | CDAI | |
|  | | Disease relapse or worsening | | Withdrawal rates for disease progression or lack of efficacy | |  | |
|  | | Response | | CDAI 70 | | CDAI | |
|  | | Response | | Change in CDAI score | | CDAI | |
|  | | Remission | | CDAI <150 and no increased or new corticosteroids, immunosuppressives or surgery | | CDAI | |
| Hommes 2002 | | Response | | CDAI 70 and reduction of >=25% from baseline score | | CDAI | |
|  | | Remission | | CDAI <150 | | CDAI | |
| Arnold 2002 | | Remission | | CDAI <150 | | CDAI | |
| Sakurai 2002 | | Remission | | CDAI 100 or reduction of 40% from baseline score | | CDAI | |
| Ardizzone 2003 | | Corticosteroid sparing | | Mean cumulative steroid dose | |  | |
|  | | Corticosteroid-free remission | | CDAI <150 and withdrawal of corticosteroids | | CDAI | |
|  | | Fistula remission | | Closure of draining enterocutaneous and perianal fistulas | |  | |
|  | | Response | | Change in CDAI score | | CDAI | |
| Ghosh 2003 | | Response | | CDAI 70 | | CDAI | |
|  | | Remission | | CDAI <150 | | CDAI | |
| Ito 2004 | | Remission | | CDAI <150 | | CDAI | |
|  | | Response | | CDAI 70 | | CDAI | |
| Joos 2004 | | Remission | | CDAI <150 | | CDAI | |
|  | | Response | | CDAI 150-160 (near remission) | | CDAI | |
| Herfarth 2004 | | Remission | | CDAI <150 | | CDAI | |
|  | | Response | | Change in CDAI score | | CDAI | |
| Mannon 2004 | | Remission | | CDAI <150 | | CDAI | |
|  | | Response | | CDAI 100 | | CDAI | |
| Sandborn 2004 | | Remission | | CDAI <150 | | CDAI | |
|  | | Fistula remission | | Closure of all draining fistulas | |  | |
|  | | Fistula response | | Reduction of 50% in draining fistula | |  | |
|  | | Response | | CDAI 100 | | CDAI | |
|  | | Response | | CDAI 70 | | CDAI | |
|  | | Response | | Mean CDAI score | | CDAI | |
| West 2004** | | Response | | Change in PDAI score | | PDAI | |
|  | | Fistula response | | Improvement on 3D-HPUS | | 3D-diagnostic ultrasound system (3D-HPUS) | |
|  | | Fistula response | | 50% reduction in draining fistula | |  | |
| Winter 2004 | | Response | | CDAI 100 | | CDAI | |
|  | | Response | | CDAI 70 | | CDAI | |
|  | | Response | | CDAI score | | CDAI | |
|  | | Remission | | CDAI <150 | | CDAI | |
| Schreiber 2005 | | Remission | | CDAI <150 | | CDAI | |
|  | | Response | | CDAI 100 | | CDAI | |
| Lomer 2005 | | Response | | CDAI decrease >=60 from baseline | | CDAI | |
|  | | Response | | CDAI score | | CDAI | |
|  | | Response | | Change in VHAI score | | VHAI | |
|  | | Remission | | CDAI <150 | | CDAI | |
| Korzenik 2005 | | Response | | CDAI 100 | | CDAI | |
|  | | Response | | CDAI 70 | | CDAI | |
|  | | Remission | | CDAI <150 | | CDAI | |
| Reinsich 2006 | | Remission | | CDAI <150 | | CDAI | |
| Margalit 2006 | | Remission | | CDAI <150 | | CDAI | |
|  | | Response | | CDAI 100 | | CDAI | |
| Prantera 2006 | | Remission | | CDAI <150 | | CDAI | |
|  | | Disease relapse or worsening | | Need for additional therapy or surgery | |  | |
|  | | Disease relapse or worsening | | CDAI increase of >100 | | CDAI | |
|  | | Response | | CDAI 70 | | CDAI | |
| Schroder 2006 | | Remission | | CDAI <150 | | CDAI | |
|  | | Corticosteroid sparing | | Mean daily prednisolone dose | |  | |
| Hommes 2006 | | Remission | | CDAI <150 | | CDAI | |
|  | | Response | | CDAI 100 | | CDAI | |
| Hanauer 2006 | | Remission | | CDAI <150 | | CDAI | |
|  | | Response | | CDAI 100 | | CDAI | |
|  | | Response | | CDAI 70 | | CDAI | |
| Lemann 2006 | | Corticosteroid sparing | | Median cumulative dose of prednisone | |  | |
|  | | Corticosteroid-free remission | | CDAI <150 and withdrawal of corticosteroids | | CDAI | |
| Rutgeerts 2006 | | Disease relapse or worsening | | Need for additional therapy | |  | |
|  | | Response | | Mean CDAI score | | CDAI | |
|  | | Remission | | CDAI <150 | | CDAI | |
| Screiber 2006 | | Fistula response | | Reduction of 50% in draining fistula | |  | |
|  | | Response | | CDAI 70 | | CDAI | |
|  | | Response | | Change in CDAI from baseline | | CDAI | |
|  | | Remission | | CDAI <150 | | CDAI | |
| Herrlinger 2006 | | Response | | CDAI 100 | | CDAI | |
|  | | Remission | | CDAI <150 | | CDAI | |
| Reinshagen 2007 | | Corticosteroid-free remission | | CDAI <150 and withdrawal of corticosteroids | | CDAI | |
| Mansfield 2007 | | Remission | | CDAI <150 and CDAI 100 | | CDAI | |
|  | | Response | | CDAI 70 | | CDAI | |
|  | | Response | | Mean change in CDAI score | | CDAI | |
| Sandborn 2007 | | Remission | | CDAI <150 | | CDAI | |
|  | | Fistula response | | 50% reduction in draining fistula | |  | |
|  | | Fistula remission | | Closure of all active draining fistulas at baseline | |  | |
|  | | Response | | CDAI 100 | | CDAI | |
|  | | Response | | CDAI 70 | | CDAI | |
|  | | Response | | Change in CDAI from baseline | | CDAI | |
| Hafer 2007 | | Remission | | CDAI <150 | | CDAI | |
|  | | Response | | CDAI 100 | | CDAI | |
|  | | Response | | Change in SAI score | | SAI | |
| Targan 2007 | | Sustained remission | | CDAI <150 over a four week period | | CDAI | |
|  | | Sustained response | | CDAI decrease of >=70 for a four week period | | CDAI | |
|  | | Remission | | CDAI <150 | | CDAI | |
|  | | Response | | CDAI 70 | | CDAI | |
| Omer 2007 | | Corticosteroid sparing | | Average dose of corticosteroids | |  | |
|  | | Response | | CDAI 70 or reduction of >=30% from baseline score | | CDAI | |
| Hart 2007** | | Response | | Physician assessment of improvement of ulcers | |  | |
|  | | Fistula response | | 50% reduction in draining fistula | |  | |
|  | | Sustained fistula remission | | Maintenance of fistula remision for at least 4 weeks | |  | |
|  | | Complete response | | Physician assessment of complete resolution of all ulcers | |  | |
| Sandborn 2008 | | Remission | | CDAI <150 | | CDAI | |
|  | | Response | | CDAI 100 | | CDAI | |
|  | | Response | | CDAI 70 or >=25% | | CDAI | |
| Leiper 2008 | | Remission | | CDAI <150 and CDAI 70 | | CDAI | |
|  | | Remission | | HBI <=4 | | HBI | |
|  | | Response | | Change in VHAI score | | VHAI | |
| D’Haens 2008 | | Corticosteroid sparing | | Daily dose of methylprednisolone | |  | |
|  | | Corticosteroid-free remission | | CDAI <150, withdrawal of corticosteroids and no surgery | | CDAI | |
|  | | Disease relapse or worsening | | proportion given infliximab, methylprednisolone and antimetabolites | |  | |
|  | | Disease relapse or worsening | | CDAI increase of >=50 points | | CDAI | |
|  | | Response | | Mean CDAI score | | CDAI | |
| Fukuda 2008** | | Fistula response | | 50% reduction in draining fistula | |  | |
|  | | Fistula remission | | Closure of all active draining fistulas at baseline | |  | |
| Feagan 2008 | | Disease relapse or worsening | | Worsening clinical status and need for additional therapy | | PGA | |
|  | | Disease relapse or worsening | | Worsening clinical status and CDAI increase of >=100 | | CDAI and PGA | |
|  | | Response | | CDAI 100 | | CDAI | |
|  | | Response | | CDAI 70 | | CDAI | |
|  | | Remission | | CDAI <150 | | CDAI | |
| Thia 2009** | | Response | | Change in CDAI score | | CDAI | |
|  | | Response | | Change in PDAI score | | PDAI | |
|  | | Fistula remission | | Closure of all active draining anal fistulas at baseline | |  | |
|  | | Fistula response | | Reduction of 50% in draining fistula | |  | |
|  | | Fistula response | | Change in mean PGA score of fistula activity | | PGA | |
|  | | Sustained fistula remission | | Maintenance of fistula remision for at least 4 weeks | |  | |
| Dotan 2010 | | Remission | | CDAI <150 | | CDAI | |
|  | | Response | | CDAI 70 | | CDAI | |
|  | | Response | | Change in Median CDAI | | CDAI | |
|  | | Response | | Mean CDAI score | | CDAI | |
|  | | Response | | Median CDAI score | | CDAI | |
| Steed 2010 | | Remission | | CDAI <150 or a reduction of >=75 | | CDAI | |
| Van der Woude 2010 | | Remission | | CDAI <150 | | CDAI | |
|  | | Response | | CDAI 100 | | CDAI | |
| Maeda 2010 | | Response | | Change in component of PDAI from baseline: degree of induration | | PDAI | |
|  | | Response | | Change in component of PDAI from baseline: discharge | | PDAI | |
|  | | Response | | Change in component of PDAI from baseline: pain / restriction of activities | | PDAI | |
|  | | Response | | Change in component of PDAI from baseline: type of perianal disease | | PDAI | |
|  | | Response | | Perianal Disease Activity Index (PDAI) - restriction of sexual activity | | PDAI | |
|  | | Response | | Change in PDAI score | | PDAI | |
| Sands 2010 | | Remission | | CDAI <150 | | CDAI | |
|  | | Response | | CDAI 100 | | CDAI | |
| Buchman 2010 | | Response | | CDAI 100 | | CDAI | |
|  | | Remission | | CDAI <150 | | CDAI | |
| Krebs 2010 | | Response | | CDAI 70 or reduction of >=30% from baseline score | | CDAI | |
| Tromm 2011 | | Remission | | CDAI <150 | | CDAI | |
|  | | Remission | | PGA of therapeutic success | | PGA | |
|  | | Response | | CDAI 100 | | CDAI | |
|  | | Response | | CDAI 70 | | CDAI | |
|  | | Response | | PGA of therapeutic benefit (category 1, 2, 3 or 4) | | PGA | |
| Sandborn 2011 | | Remission | | CDAI <150 | | CDAI | |
|  | | Response | | CDAI 100 | | CDAI | |
|  | | Response | | CDAI score | | CDAI | |
|  | | Response | | Change in HBI from baseline | | HBI | |
| Benjamin 2011 | | Remission | | CDAI <150 | | CDAI | |
|  | | Response | | CDAI 70 | | CDAI | |
|  | | Response | | Change in CDAI score | | CDAI | |
| Smith 2011 | | Response | | CDAI 70 | | CDAI | |
| Prantera 2012 | | Disease relapse or worsening | | Need for additional therapy or surgery | |  | |
|  | | Disease relapse or worsening | | CDAI fail to decrease by at least 70 points from baseline | | CDAI | |
|  | | Disease relapse or worsening | | CDAI increase of >100 from baseline | | CDAI | |
|  | | Sustained remission | | CDAI <150 sustained for the length of the study | | CDAI | |
|  | | Remission | | CDAI <150 | | CDAI | |
|  | | Response | | CDAI 100 | | CDAI | |
| Hueber 2012 | | Remission | | CDAI <150 | | CDAI | |
|  | | Response | | CDAI 100 | | CDAI | |
|  | | Response | | Change in CDAI score | | CDAI | |
| Sands 2013 | | Remission | | CDAI <150 | | CDAI | |
|  | | Response | | CDAI 100 | | CDAI | |
|  | | Response | | Change in CDAI from baseline | | CDAI | |
| Naftali 2013 | | Remission | | CDAI <150 | | CDAI | |
|  | | Response | | CDAI 100 | | CDAI | |
| Suzuki 2013 | | Remission | | CDAI <150 | | CDAI | |
|  | | Response | | Change in CDAI score | | CDAI | |
| Brotherton 2014 | | Response | | Mean pHBI score | | pHBI | |
| Sandborn 2014 | | Remission | | CDAI <150 | | CDAI | |
|  | | Response | | CDAI 100 | | CDAI | |
|  | | Response | | CDAI 70 | | CDAI | |
| Dewint 2014** | | Fistula remission | | 100% reduction in draining fistula | |  | |
|  | | Fistula response | | 50% reduction in draining fistula | |  | |
| Reinisch 2014** | | Response | | Change in CDAI score | | CDAI | |
|  | | Fistula remission | | Closure of all draining fistulas | |  | |
|  | | Fistula response | | Reduction of 50% in draining fistula | |  | |
| Sands 2014 | | Remission | | CDAI <150 | | CDAI | |
|  | | Response | | CDAI 100 | | CDAI | |
| Dignass 2014 | | Remission | | CDAI <150 | | CDAI | |
|  | | Response | | Mean CDAI score | | CDAI | |
|  | | Response | | Change in PGA score | | PGA | |
| Bao 2014 | | Remission | | CDAI <150 | | CDAI | |
|  | | Disease relapse or worsening | | CDAI decreased by <70 or increased CDAI | | CDAI | |
|  | | Response | | CDAI 70 | | CDAI | |
| D’Haens 2015 | | Remission | | CDAI <150 and no treatment failures | | CDAI | |
|  | | Response | | CDAI 100 | | CDAI | |
|  | | Response | | CDAI 70 | | CDAI | |
| Monteleone 2015 | | Remission | | CDAI <150 | | CDAI | |
|  | | Sustained remission | | CDAI <150 maintained for at least 2 weeks | | CDAI | |
|  | | Response | | CDAI 100 | | CDAI | |
|  | | Response | | CDAI 70 | | CDAI | |
|  | | Response | | Change in median CDAI | | CDAI | |
|  | | Response | | Mean CDAI score | | CDAI | |
| Vande Casteele 2015 | | Disease relapse or worsening | | Need for additional therapy | |  | |
|  | | Sustained remission | | HBI <=4 and CRP concentration of <=5mg/L throughout study | | HBI | |
|  | | Remission | | HBI <=4 and CRP of <=5mg/L | | HBI | |
| **Surgical induction (n=6)** | |  | |  | |  | |
| Maartense 2006 | | Post-operative recovery | | Duration of hospital stay (days) | |  | |
|  | | Post-operative recovery | | Morphine requirement | |  | |
| East 2007 | | Disease relapse or worsening | | Time to repeat dilation or surgery | |  | |
| Mcleod 2009 | | Combined clinical and endoscopic recurrence | | RES >=2 and need for additional therapy or surgery | | Rutgeerts endoscopic score | |
| Grimaud 2010** | | Response | | Occurrence of perianal abscess | |  | |
|  | | Fistula remission | | Absence of draining fistula, absence of perianal pain and absence of perianal abscess | |  | |
|  | | Fistula response | | Closure of 50% or more of draining anal fistulas | |  | |
| Zurbuchen 2013 | | Recurrence | | Need for additional surgery | |  | |
|  | | Post-operative recovery | | Duration of hospital stay (days) | |  | |
|  | | Post-operative recovery | | Time (days) to first postoperative stool | |  | |
| Molendijk 2015** | | Fistula response | | Reduced number of draining fistulas | |  | |
|  | | Response | | MRI evaluation of fistula tracts | |  | |
|  | | Response | | Change in CDAI score | | CDAI | |
|  | | Response | | Change in PDAI score | | PDAI | |
| **Maintenance (n=65)** | | | | | | | |
| **Maintenance studies of medically induced remission (n=47)** | | | | | | | |
| Singleton 1979 | | Response | | Change in CDAI score | | CDAI | |
|  | | Disease relapse or worsening | | Withdrawn early for severe exacerbation or drug toxicity | |  | |
|  | | Disease relapse or worsening | | Withdrawn early for surgery | |  | |
|  | | Disease relapse or worsening | | CDAI > 150 or >40% of initial CDAI | | CDAI | |
|  | | Sustained remission | | CDAI <150 for the length of the study | | CDAI | |
|  | | Treatment compliance | | Pill count | |  | |
|  | | Remission | | CDAI <150 | | CDAI | |
| Malchow 1984 | | Disease relapse or worsening | | Development of a new abscess | |  | |
|  | | Disease relapse or worsening | | Pending surgery for complication of Crohn's | |  | |
|  | | Disease relapse or worsening | | CDAI >150 requiring repetition of acute phase treatment | | CDAI | |
|  | | Disease relapse or worsening | | CDAI increase of >100, no change or minimal reduction <60 | | CDAI | |
| Bresci 1994 | | Disease relapse or worsening | | CDAI >150 or increase >=100 | | CDAI | |
| Feagan 1994 | | Response | | Mean CDAI score | | CDAI | |
|  | | Corticosteroid sparing | | Mean dose of prednisone and 5-aminosalicylates | |  | |
|  | | Disease relapse or worsening | | CDAI increase of >=100 | | CDAI | |
| Schreiber 1994 | | Disease relapse or worsening | | CDAI >150 and an increase of >=100 points | | CDAI | |
|  | | Sustained remission | | CDAI <150 maintained for 12 months | | CDAI | |
| Stange 1995 | | Response | | CDAI between 150 and 200 | | CDAI | |
|  | | Response | | HBI score | | HBI | |
|  | | Response | | Change in Present score | | Present score | |
|  | | Response | | Change in VHAI score | | VHAI | |
|  | | Disease relapse or worsening | | CDAI >200 | | CDAI | |
|  | | Remission | | CDAI <150 | | CDAI | |
| Belluzzi 1996 | | Disease relapse or worsening | | CDAI >150 and an increase of >=100 points | | CDAI | |
| Greenberg 1996 | | Disease relapse or worsening | | Need for additional therapy or surgery | |  | |
|  | | Disease relapse or worsening | | CDAI >150 and an increase of >=60 points | | CDAI | |
| Sutherland 1997 | | Disease relapse or worsening | | Investigator opinion of relpase | |  | |
|  | | Disease relapse or worsening | | Need for hospitalisation due to worsening Crohn's | |  | |
|  | | Disease relapse or worsening | | Need for introduction of corticosteroids | |  | |
|  | | Disease relapse or worsening | | CDAI >150 and an increase of >=60 points | | CDAI | |
| Ferguson 1998 | | Response | | Mean CDAI score | | CDAI | |
|  | | Disease relapse or worsening | | CDAI >150 and an increase of >=60 points | | CDAI | |
| Arora 1999 | | Disease relapse or worsening | | Withdrew from study due to disease flare or failure to reduce prednisone dose | |  | |
| Guslandi 2000 | | Disease relapse or worsening | | CDAI >150 and an increase of >=100 points | | CDAI | |
| Green 2001 | | Disease relapse or worsening | | Moderate to severe symptoms with either high steroid dose or CDAI >200 | |  | |
| Mahmud 2001 | | Disease relapse or worsening | | Need for additional therapy or surgery | |  | |
|  | | Disease relapse or worsening | | CDAI >150 and an increase of >=60 points | | CDAI | |
| Cortot 2001 | | Response | | Change in CDAI score | | CDAI | |
|  | | Disease relapse or worsening | | Crohn's Disease Activity Index (CDAI) | | CDAI | |
| Hanauer 2002 | | Disease relapse or worsening | | CDAI score >175 and an increase of >=70 points or >=35% | | CDAI | |
|  | | Remission | | CDAI <150 | | CDAI | |
| Mantzaris 2003 | | Response | | Change in CDAI score | | CDAI | |
| Mantzaris 2003 | | Disease relapse or worsening | | CDAI >150 and an increase of >=100 points | | CDAI | |
| Keller 2004 | | Disease relapse or worsening | | Failure of any drug treatment including immunosupressants, with or without surgery | | ECCDS | |
|  | | Disease relapse or worsening | | Failure of standard drug therapy, but effective immunosuppressive therapy | | ECCDS | |
|  | | Remission | | Relapse-free course | | ECCDS | |
| Sands 2004** | | Response | | CDAI 70 or by 25% from start of >220 | | CDAI | |
|  | | Response | | CDAI score | | CDAI | |
|  | | Disease relapse or worsening | | Study discontinuation due to perceived inefficacy | |  | |
|  | | Disease relapse or worsening | | Need for additional therapy or surgery | |  | |
|  | | Disease relapse or worsening | | Recurrence of draining fistula | |  | |
| Schultz 2004 | | Disease relapse or worsening | | CDAI increased by >100 points | | CDAI | |
|  | | Sustained remission | | CDAI <150 sustained for the length of the study | | CDAI | |
| Vilien 2004 | | Disease relapse or worsening | | Need for additional therapy or surgery | |  | |
|  | | Disease relapse or worsening | | CDAI >150 or an increase of >=75 points | | CDAI | |
| Lemann 2005 | | Disease relapse or worsening | | Need for surgery | |  | |
|  | | Disease relapse or worsening | | CDAI >250 or CDAI 150-250 and an increase of >=75 points from baseline | | CDAI | |
| Feagan 2005 | | Response | | Median CDAI score | | CDAI | |
|  | | Corticosteroid sparing | | Withdrawal of corticosteroids, no flare (CDAI >=220) and no study withdrawal | | CDAI | |
|  | | Disease relapse or worsening | | CDAI >=220 | | CDAI | |
|  | | Response | | Mean CDAI score | | CDAI | |
| Hanauer 2005 | | Response | | Change in CDAI score | | CDAI | |
|  | | Disease relapse or worsening | | CDAI >150 and an increase of >=60 points or clinical deterioration | | CDAI | |
| Sandborn 2005 | | Sustained remission | | CDAI <150 sustained for the length of the study | | CDAI | |
|  | | Sustained response | | CDAI 70 sustained for the length of the study | | CDAI | |
|  | | Remission | | CDAI <150 | | CDAI | |
| Feagan 2006 | | Response | | Mean CDAI score | | CDAI | |
|  | | Corticosteroid sparing | | Withdrawal of corticosteroids and no flare (CDAI >220) | | CDAI | |
|  | | Disease relapse or worsening | | Increase in steroids, total steroids taken | |  | |
|  | | Fistula remission | | Closure of all draining fistulas | |  | |
|  | | Fistula response | | Reduction of 50% in draining fistula | |  | |
| Takagi 2006 | | Disease relapse or worsening | | Need for additional therapy | |  | |
|  | | Disease relapse or worsening | | CDAI >200 | | CDAI | |
| Colombel 2007 | | Remission | | CDAI <150 | | CDAI | |
|  | | Corticosteroid-free remission | | CDAI <150 and withdrawal of corticosteroids | | CDAI | |
|  | | Sustained corticosteroid-free remission | | CDAI<150 and able to discontinue corticosteroid use for >=90days | | CDAI | |
|  | | Fistula remission | | Closure of all fistulas that were draining at screening and baseline visits | |  | |
|  | | Response | | CDAI 100 | | CDAI | |
|  | | Response | | CDAI 70 | | CDAI | |
| Sandborn 2007 | | Remission | | CDAI <150 | | CDAI | |
|  | | Response | | CDAI 100 | | CDAI | |
|  | | Response | | CDAI 70 | | CDAI | |
|  | | Corticosteroid-free remission | | CDAI <150 and withdrawal of corticosteroids | | CDAI | |
| Ng 2007 | | Response | | HBI score | | HBI | |
| Schreiber 2007 | | Remission | | CDAI <150 | | CDAI | |
|  | | Response | | CDAI 100 | | CDAI | |
| De Jong 2007 | | Remission | | CDAI <150 | | CDAI | |
|  | | Disease relapse or worsening | | CDAI >150 and an increase of >=60 points | | CDAI | |
| Selby 2007 | | Disease relapse or worsening | | Need for additional therapy | |  | |
|  | | Disease relapse or worsening | | CDAI >150 and an increase of >=60 points | | CDAI | |
|  | | Remission | | CDAI <150 | | CDAI | |
| Feagan 2008 | | Response | | Change in CDAI score | | CDAI | |
|  | | Disease relapse or worsening | | Need for additional therapy or surgery | |  | |
|  | | Disease relapse or worsening | | CDAI >150 and an increase of >=70 points | | CDAI | |
| Mantzaris 2009 | | Response | | Mean CDAI score | | CDAI | |
|  | | Disease relapse or worsening | | CDAI >150 and an increase of >=100 points | | CDAI | |
| Rossi 2009 | | Response | | Change in CDAI from baseline | | CDAI | |
|  | | Response | | Number of fistulas including new ones and closure of existing ones | |  | |
|  | | Disease relapse or worsening | | CDAI >220 and increase of >=70 and need for additional therapy | | CDAI | |
| Valentine 2009 | | Corticosteroid-free response | | CDAI decrease of >=100 points and withdrawal of corticosteroids | | CDAI | |
|  | | Corticosteroid-free remission | | CDAI <150 and withdrawal of corticosteroids | | CDAI | |
|  | | Corticosteroid-free remission | | CDAI score <150 and >=25% decrease and withdrawal of corticosteroids | | CDAI | |
|  | | Corticosteroid-free remission | | CDAI score <150 and 100 point decrease and withdrawal of corticosteroids | | CDAI | |
|  | | Disease relapse or worsening | | Mean daily corticosteroid use at study withdrawal | |  | |
|  | | Disease relapse or worsening | | Study withdrawal | |  | |
|  | | Response | | CDAI 100 | | CDAI | |
|  | | Response | | Change in mean CDAI | | CDAI | |
|  | | Remission | | CDAI <150 | | CDAI | |
| Sandborn 2010 | | Remission | | CDAI <150 | | CDAI | |
|  | | Response | | CDAI 100 | | CDAI | |
|  | | Response | | CDAI 70 | | CDAI | |
|  | | Response | | Mean CDAI score | | CDAI | |
| Jorgensen 2010 | | Disease relapse or worsening | | CDAI >150 and an increase of >=70 points | | CDAI | |
| Prantera 2011 | | Response | | Change in CDAI score | | CDAI | |
|  | | Disease relapse or worsening | | Study withdrawal due to disease deterioration | |  | |
|  | | Disease relapse or worsening | | CDAI >150 and an increase of >=60 points | | CDAI | |
| Holtmeier 2011 | | Response | | Mean change in CDAI score | | CDAI | |
|  | | Disease relapse or worsening | | Clinical need for introduction of corticosteroid therapy or hospitalisation for flare-up | |  | |
|  | | Disease relapse or worsening | | Investigator opinion of relpase | |  | |
|  | | Disease relapse or worsening | | Surgical intervention for stenosis, fistula or abscesses | |  | |
|  | | Disease relapse or worsening | | CDAI >150 and an increase of >=70 points | | CDAI | |
|  | | Sustained remission | | CDAI <150 maintained for 12 months | | CDAI | |
|  | | Treatment compliance | | Pill count | |  | |
| Watanabe 2012 | | Remission | | CDAI <150 | | CDAI | |
|  | | Response | | CDAI 100 | | CDAI | |
|  | | Response | | CDAI 70 | | CDAI | |
|  | | Response | | Change in CDAI from baseline | | CDAI | |
|  | | Response | | Change in IOIBD from baseline | | IOIBD | |
| Keshav 2013 | | Remission | | CDAI <150 | | CDAI | |
|  | | Sustained remission | | CDAI <150 sustained for the length of the study | | CDAI | |
|  | | Response | | CDAI 100 | | CDAI | |
|  | | Sustained response | | CDAI 70 sustained for the length of the study | | CDAI | |
|  | | Response | | CDAI 70 | | CDAI | |
| Bourreile 2013 | | Response | | Mean CDAI score | | CDAI | |
|  | | Disease relapse or worsening | | Need for additional therapy or surgery | |  | |
|  | | Disease relapse or worsening | | Crohn's Disease Activity Index (CDAI) | | CDAI | |
| Jigaranu 2014 | | Sustained remission | | CDAI <150 sustained for the length of the study | | CDAI | |
|  | | Sustained response | | CDAI 100 sustained for the length of the study | | CDAI | |
| Feagan 2014 | | Sustained corticosteroid-free remission | | CDAI <150 and withdrawal of prednisone for length of trial | | CDAI | |
|  | | Corticosteroid-free remission | | CDAI <150 and withdrawal of corticosteroids | | CDAI | |
|  | | Disease relapse or worsening | | Failure to achieve CDAI<150 and total withdrawal of prednisone or failure to maintain CDAI <150 until the end of the study | | CDAI | |
|  | | Disease relapse or worsening | | Failure to achieve CDAI<150 and total withdrawal of prednisone or failure to maintain CDAI <150 until the end of the study | | CDAI | |
|  | | Response | | Mean change in CDAI score | | CDAI | |
| Wenzl 2015 | | Disease relapse or worsening | | Development of one or more new fistula | |  | |
|  | | Disease relapse or worsening | | Need for additional surgery | |  | |
|  | | Disease relapse or worsening | | Need for steroids or anti-TNF | |  | |
|  | | Disease relapse or worsening | | CDAI >150 and an increase of >=60 points | | CDAI | |
|  | | Disease relapse or worsening | | PDAI increase of >4 points | | PDAI | |
|  | | Response | | CDAI score | | CDAI | |
| **Maintenance studies of surgically induced remission (n=18)** | | | | | | | |
| McLeod 1995 | | Combined clinical and endoscopic recurrence | | Investigator assessment of disease activity and radiological or endoscopic evidence | |  | |
| Ewe 1999 | | Response | | Median CDAI score | | CDAI | |
|  | | Recurrence | | Signs and symptoms characteristic of Crohn's | |  | |
|  | | Recurrence | | CDAI >200 and rise of 60 points | | CDAI | |
| Hellers 1999 | | Response | | Mean CDAI score | | CDAI | |
|  | | Response | | Change in PGA score | | PGA | |
| Lochs 2000 | | Recurrence | | Development of one or more new fistula or septic complication | |  | |
|  | | Recurrence | | Need for additional surgery | |  | |
|  | | Recurrence | | CDAI >250 | | CDAI | |
|  | | Recurrence | | CDAI >200 and rise of 60 points | | CDAI | |
| Colombel 2001 | | Recurrence | | Need for additional therapy | |  | |
| Prantera 2002 | | Recurrence | | CDAI >150 | | CDAI | |
| Caprilli 2003 | | Recurrence | | CDAI >150 | | CDAI | |
| Hanauer 2004 | | Recurrence | | Clinical recurrence grading scale | |  | |
| Marteau 2006 | | Recurrence | | CDAI >=200 | | CDAI | |
| D’Haens 2008 | | Recurrence | | CDAI >250 | | CDAI | |
| Regueiro 2009 | | Recurrence | | CDAI >200 | | CDAI | |
| Reinisch 2010 | | Response | | Mean CDAI change from baseline | | CDAI | |
|  | | Recurrence | | CDAI >=200 and an increase of >=60 | | CDAI | |
| Savarino 2013 | | Recurrence | | CDAI >200 | | CDAI | |
| Herfarth 2013 | | Recurrence | | HBI score >=5 or an increase of 3 points since previous visit | | HBI | |
| Ren 2013 | | Response | | Change in CDAI score | | CDAI | |
|  | | Combined clinical and endoscopic recurrence | | RES >=2 and either symptoms and symptoms of Crohn's or CDAI >150, needing therapy or surgery | | Rutgeerts endoscopic score and CDAI | |
| Armuzzi 2013 | | Recurrence | | HBI >=8 | | HBI | |
| Fedorak 2015 | | Response | | Mean CDAI score | | CDAI | |
| Zhu 2015 | | Recurrence | | Need for additional therapy or surgery | |  | |
| Note: CDAI, Crohn’s disease activity index; IOIBD, International Organisation of Inflammatory Bowel Disease; HBI, Harvey Bradshaw Index; ESR, erythrocyte sedimentation rate; CRP, C-reactive protein; PDAI, perianal disease activity index; **, study involved only patients with fistula.  ** Trials in fistula patients | | | | | | | |

Supplementary Table 7: Primary and Secondary Endoscopic Efficacy Outcomes in Crohn's Disease Randomised Controlled Trials

| **Reference** | | **Outcome** | **Outcome measure** | **Outcome measurement tool** |
| --- | --- | --- | --- | --- |
| **Induction (n=13)** | |  |  |  |
| **Medical induction (n=10)** | | | | |
| Schreiber 2000 | | Endoscopic response | Change in CDEIS score from baseline | CDEIS |
| Reinsich 2006 | | Endoscopic response | Change in CDEIS score from baseline | CDEIS |
| Lemann 2006 | | Endoscopic response | Change in CDEIS score from baseline | CDEIS |
| Schreiber 2006 | | Endoscopic response | Change in CDEIS score from baseline | CDEIS |
| D’Haens 2008 | | Endoscopic mucosal healing | No ulcers identified | SES-CD |
|  | | Endoscopic response | Mean SES-CD severity score | SES-CD |
| Van der Woude 2010 | | Endoscopic response | Mean change in CDEIS score | CDEIS |
| Sands 2010 | | Endoscopic response | Change in CDEIS score from baseline | CDEIS |
|  | | Endoscopic response | Change in SES-CD score from baseline | SES-CD |
| Smith 2011 | | Endoscopic remission | CDEIS score <6 | CDEIS |
|  | | Endoscopic response | CDEIS score <3 | CDEIS |
|  | | Endoscopic response | CDEIS score decrease by >=5 | CDEIS |
|  | | Endoscopic response | Change in CDEIS score | CDEIS |
| Dignass 2014 | | Endoscopic mucosal healing | Complete mucosal healing: SES-CD score of 0 | SES-CD |
| Bao 2014 | | Endoscopic response | CDEIS score | CDEIS |
| **Surgical induction (n=3)** | | | | |
| Mcleod 2009 | | Endoscopic recurrence | Rutgeerts endoscopic score >=2 | Rutgeerts endoscopic score |
| Zurbuchen 2013 | | Endoscopic recurrence | Rate of endoscopic recurrence | Rutgeerts endoscopic score |
| Molendijk 2015** | | Endoscopic response | Change in CDEIS score | CDEIS |
|  | | Endoscopic response | Change in SES-CD score | SES-CD |
| **Maintenance (n=22)** | |  |  |  |
| **Maintenance studies of medically induced remission (n=3)** | | | | |
| Malchow 1984 | | Endoscopic recurrence | Endoscopic or radiological results document a worsening of condition |  |
| Selby 2007 | | Endoscopic response | Change in CDEIS score | CDEIS |
| Mantzaris 2009 | | Endoscopic mucosal healing | Change in endoscopic category of mucosal healing | D'Haens endoscopic categories |
|  | | Endoscopic response | Change in CDEIS score | CDEIS |
| **Maintenance studies of surgically induced remission (n=19)** | | | | |
| Brignola 1995 | | Endoscopic recurrence | Rutgeerts endoscopic score >2 | Rutgeerts endoscopic score |
|  | | Endoscopic recurrence | Rutgeerts endoscopic score >2 or radiological documentation of recurrence | Rutgeerts endoscopic score |
|  | | Endoscopic response | Mean Rutgeerts endoscopic score | Rutgeerts endoscopic score |
| McLeod 1995 | | Endoscopic recurrence | Rate of endoscopic or radiological recurrence |  |
| Ewe 1999 | | Endoscopic recurrence | Rutgeerts endoscopic score >=2 | Rutgeerts endoscopic score |
| Hellers 1999 | | Endoscopic recurrence | Rutgeerts endoscopic score >=2 | Rutgeerts endoscopic score |
| Lochs 2000 | | Endoscopic recurrence | Rutgeerts score >=2 | Rutgeerts endoscopic score |
| Colombel 2001 | | Endoscopic recurrence | Rutgeerts endoscopic score >0 | Rutgeerts endoscopic score |
| Prantera 2002 | | Endoscopic recurrence | Rutgeerts endoscopic score >=2 | Rutgeerts endoscopic score |
|  | | Endoscopic recurrence | Rutgeerts endoscopic score >2 | Rutgeerts endoscopic score |
| Caprilli 2003 | | Endoscopic recurrence | Rutgeerts endoscopic score >0 | Rutgeerts endoscopic score |
|  | | Endoscopic recurrence | Rutgeerts endoscopic score >2 | Rutgeerts endoscopic score |
| Hanauer 2004 | | Endoscopic recurrence | Radiographic recurrence grading scale >=2 | Radiographic grading scale |
|  | | Endoscopic recurrence | Rutgeerts endoscopic score >=2 | Rutgeerts endoscopic score |
| Marteau 2006 | | Endoscopic recurrence | Colonic lesions endoscopic score >1 |  |
|  | | Endoscopic recurrence | Rutgeerts endoscopic score >1 | Rutgeerts endoscopic score |
|  | | Endoscopic response | Maximum colonic lesions endoscopic score |  |
|  | | Endoscopic response | Maximum Rutgeerts endoscopic score | Rutgeerts endoscopic score |
| D’Haens 2008 | | Endoscopic recurrence | Rutgeerts endoscopic score >=2 | Rutgeerts endoscopic score |
|  | | Endoscopic recurrence | Rutgeerts endoscopic score >2 | Rutgeerts endoscopic score |
| Regueiro 2009 | | Endoscopic recurrence | Rutgeerts endoscopic score >=2 | Rutgeerts endoscopic score |
| Reinisch 2010 | | Endoscopic mucosal healing | Median improvement in CDEIS score | CDEIS |
|  | | Endoscopic response | >=1 point improvement in Rutgeerts score | Rutgeerts endoscopic score |
| Savarino 2013 | | Endoscopic recurrence | Rutgeerts endoscopic score >=2 | Rutgeerts endoscopic score |
| Herfarth 2013 | | Endoscopic recurrence | Marteau score >= c2 | Marteau endoscopic score |
|  | | Endoscopic recurrence | Rutgeerts score >=2 | Rutgeerts endoscopic score |
| Ren 2013 | | Endoscopic recurrence | Rutgeerts endoscopic score >=2 | Rutgeerts endoscopic score |
| Ren 2013 | | Endoscopic response | Change in Rutgeerts endoscopic score | Rutgeerts endoscopic score |
| Armuzzi 2013 | | Endoscopic recurrence | Rutgeerts endoscopic score >=2 | Rutgeerts endoscopic score |
| Fedorak 2015 | | Endoscopic recurrence | Rutgeerts endoscopic score >0 | Rutgeerts endoscopic score |
|  | | Endoscopic recurrence | Rutgeerts endoscopic score >2 | Rutgeerts endoscopic score |
| Zhu 2015 | | Endoscopic recurrence | Rutgeerts endoscopic score >=2 | Rutgeerts endoscopic score |
| Note: CDEIS, Crohn's Disease Endoscopic Index of Severity; SES-CD, Simple Endoscopic Score for Crohn's disease;**, study involved only patients with fistula.  ** Trials in fistula patients | | | | |

Supplementary Table 8: Primary and Secondary Histology Efficacy Outcomes in Crohn's Disease Randomised Controlled Trials

| **Reference** | **Outcome** | **Outcome measure** | **Outcome measurement tool** |
| --- | --- | --- | --- |
| **Induction (n=5)** | | | |
| **Medical induction (n=5)** | | | |
| Schreiber 2000 | Tissue cytokine, leukocyte, receptor or gene expression | Total concentration of NFκB and IκBα cytokine |  |
| Mannon 2004 | Histologic response | D'Haens histologic score | D'Haens histological activity score |
| Mannon 2004 | Tissue cytokine, leukocyte, receptor or gene expression | Mononuclear cell secretion of Interferon-γ cytokine |  |
| Steed 2010 | Tissue cytokine, leukocyte, receptor or gene expression | Change in mucosal TNF-alpha cytokines |  |
| Smith 2011 | Histologic response | Dieleman histological score | Dieleman histological score |
| Bao 2014 | Histologic response | D'Haens histological activity score of biopsy | D'Haens histological activity score |
| **Maintenance (n=6)** | | | |
| **Maintenance studies of medically induced remission (n=1)** | | | |
| Mantzaris 2009 | Histologic remission | Average histology score (AHS) | Average histology score (AHS) |
| **Maintenance studies of surgically induced remission (n=5)** | | | |
| Ewe 1999 | Histologic recurrence | Histological score of 2 or 1 in conjuction with special findings | Histological Activity Score |
| Colombel 2001 | Histologic recurrence | D'Haens histological activity score of biopsy | D'Haens histological activity score |
| Regueiro 2009 | Histologic recurrence | D'Haens histological activity score and presence of neutrophils | D'Haens histological activity score |
| Armuzzi 2013 | Histologic recurrence | Regueiron histology score of moderate to severe activity | Regueiro histology score |
| Fedorak 2015 | Tissue cytokine, leukocyte, receptor or gene expression | Mucosal inflammatory cytokines expression |  |

Supplementary Table 9: Primary and Secondary Patient Reported Outcomes in Crohn's Disease Randomised Controlled Trials

| **Reference** | **Outcome** | **Outcome measure** | **Outcome measurement tool** |
| --- | --- | --- | --- |
| **Induction studies (n=47)** | | | |
| **Medical induction (n=45)** | | | |
| Singleton 1993 | Defecation functions | Patient diary score of diarrhea |  |
|  | Pain | Mean patient assessment of abdominal pain, diarrhea and overall well-being |  |
|  | Quality of life | Mean patient assessment of abdominal pain, diarrhea and overall well-being |  |
| Greenberg 1994 | Quality of life | Mean IBDQ score | IBDQ |
| Feagan 1995 | Quality of life | Mean IBDQ score | IBDQ |
| Sandborn 1999 | Quality of life | Mean IBDQ score | IBDQ |
| Verma 2000 | Bowel symptoms | Patient report of bowel symptoms such as diarrhea, rectal bleeding or pain |  |
| Fedorak 2000 | Quality of life | IBDQ score | IBDQ |
| Schreiber 2000 | Quality of life | Change in IBDQ from baseline | IBDQ |
|  | Quality of life | Change in SF-36 physical scale score | SF-36 |
| Leiper 2001 | Quality of life | Improvement in IBDQ score | IBDQ |
| Schreiber 2001 | Quality of life | Change in IBDQ from baseline | IBDQ |
|  | Quality of life | Median IBDQ score | IBDQ |
| Hawkes 2001 | Defecation functions | Change in stool frequency |  |
|  | Pain | Change in abdominal pain |  |
|  | Quality of life | Change in Short-IBDQ score | Short-IBDQ |
| Sandborn 2001 | Quality of life | IBDQ score | IBDQ |
| Carty 2001 | Quality of life | Patient global assessment of changes in clinical condition | Patient Global Assessment |
| Tremaine 2002 | Quality of life | Change in IBDQ from baseline | IBDQ |
|  | Quality of life | Change in SF-36 from baseline | SF-36 |
|  | Quality of life | Change in SF-36 mental component summary score from baseline | SF-36 MCS |
|  | Quality of life | Change in SF-36 physical component summary score from baseline | SF-36 PCS |
| Steinhart 2002 | Quality of life | Change in IBDQ from baseline | IBDQ |
| Ghosh 2003 | Quality of life | Median IBDQ score | IBDQ |
| Ito 2004 | Quality of life | Change in IBDQ from baseline | IBDQ |
| Joos 2004 | Quality of life | Change in IBDQ from baseline | IBDQ |
|  | Quality of life | VAS measure of general wellbeing score | Visual analogue scale |
| Sandborn 2004 | Quality of life | Mean IBDQ score | IBDQ |
| Lomer 2005 | Quality of life | IBDQ score | IBDQ |
| Korzenik 2005 | Quality of life | Change in IBDQ from baseline | IBDQ |
| Reinsich 2006 | Quality of life | Mean IBDQ score | IBDQ |
| Margalit 2006 | Quality of life | Mean IBDQ score | IBDQ |
| Prantera 2006 | Quality of life | Mean IBDQ score | IBDQ |
| Hanauer 2006 | Quality of life | Change in IBDQ from baseline | IBDQ |
| Schreiber 2006 | Quality of life | Change in IBDQ from baseline | IBDQ |
| Reinshagen 2007 | Quality of life | Mean IBDQ score | IBDQ |
| Sandborn 2007 | Quality of life | Change in IBDQ from baseline | IBDQ |
| Omer 2007 | Quality of life | Decrease in HAMD score from baseline | Hamilton Depression Scale |
| Fukuda 2008** | Defecation functions | Patient diary score of fecal consistency |  |
|  | Pain | Change in symptom scores for perianal pain |  |
|  | Quality of life | Change in symptom scores for drainage amount (patient diary) |  |
| Leiper 2008 | Quality of life | Decrease in inflammatory bowel specific Quality of Life Index | IBD Quality of Life Index |
| D’Haens 2008 | Quality of life | Mean IBDQ score | IBDQ |
| Feagan 2008 | Quality of life | Change in IBDQ from baseline | IBDQ |
| Thia 2009** | Quality of life | Change in IBDQ from baseline | IBDQ |
|  | Quality of life | Change in mean Patient Global Assessment score | Patient Global Assessment |
| Dotan 2010 | Quality of life | Change in median IBDQ | IBDQ |
|  | Quality of life | Mean IBDQ score | IBDQ |
|  | Quality of life | Median IBDQ | IBDQ |
| Maeda 2010 | Pain | Change in perianal pain (VA-Scale) from baseline | Visual analogue scale |
|  | Quality of life | Change in SF12 scores from baseline | SF-12 |
|  | Treatment acceptability | Patient global assessment of improvement (Likert scale) | Patient Global Assessment |
| Sands 2010 | Quality of life | Change IBDQ score from baseline | IBDQ |
| Buchman 2010 | Defecation functions | Mean change in number of liquid bowel movements |  |
|  | Treatment compliance | Patient diary and pill count |  |
| Krebs 2010 | Quality of life | Decrease in HAMD score from baseline | Hamilton Depression Scale |
| Sandborn 2011 | Quality of life | IBDQ score >170 | IBDQ |
| Benjamin 2011 | Quality of life | Change in IBDQ from baseline | IBDQ |
| Naftali 2013 | Quality of life | Improvement in SF-36 score of at least 50 points | SF-36 |
| Brotherton 2014 | Quality of life | Mean IBDQ score | IBDQ |
| Dignass 2014 | Quality of life | SHS value | Short Health Scale |
|  | Quality of life | Total GIQLI score | GIQLI |
| Bao 2014 | Quality of life | IBDQ score | IBDQ |
| **Surgical induction (n=2)** | | | |
| Maartense 2006 | Pain | Pain VAS score | Visual analogue scale |
|  | Pain | SF-36 bodily pain score | SF-36 bodily pain score |
|  | Quality of life | SF-36 emotional score | SF-36 |
|  | Quality of life | SF-36 General health perception score | SF-36 general health perception score |
|  | Quality of life | SF-36 mental component scale score | SF-36 MCS |
|  | Quality of life | SF-36 mental health score | SF-36 mental health score |
|  | Quality of life | SF-36 physical component scale score | SF-36 |
|  | Quality of life | SF-36 physical function score | SF-36 physical function score |
|  | Quality of life | SF-36 physical role score | SF-36 |
|  | Quality of life | SF-36 Social function score | SF-36 social function score |
|  | Quality of life | SF-36 vitality score | SF-36 vitality score |
|  | Quality of life | Total GIQLI score | GIQLI |
| Molendijk 2015** | Defecation functions | Changes in adapted Vaizey fecal incontinence score | Adapted Vaizey fecal incontinence score |
|  | Quality of life | Change in SF-36 from baseline | SF-36 |
|  |  | Changes in short IBDQ score | Short-IBDQ |
| **Maintenance studies (n=28)** | | | |
| **Maintenance studies of medically induced remission (n=24)** | | | |
| Feagan 1994 | Quality of life | Mean IBDQ score | IBDQ |
| Greenberg 1996 | Quality of life | Mean IBDQ score | IBDQ |
| Sutherland 1997 | Quality of life | Mean IBDQ score | IBDQ |
| Cortot 2001 | Quality of life | Change in IBDQ from baseline | IBDQ |
| Mantzaris 2003 | Quality of life | Change in IBDQ from baseline | IBDQ |
| Sands 2004** | Quality of life | IBDQ score | IBDQ |
| Keller 2004 | Quality of life | Mean depression BDI score | BDI - depression score |
|  | Quality of life | Mean PSKB score | PSKB |
|  | Quality of life | Mean QL score | Quality of Life instrument |
|  | Quality of life | Mean Trait anxiety STAI-X2 score | STAI-X2 - trait anxiety score |
| Feagan 2005 | Quality of life | Median IBDQ score | IBDQ |
| Feagan 2006 | Quality of life | Mean IBDQ score | IBDQ |
| Colombel 2007 | Quality of life | Change in IBDQ from baseline | IBDQ |
| Sandborn 2007 | Quality of life | Change in IBDQ from baseline | IBDQ |
| Ng 2007 | Quality of life | IBD Stress Index Score | IBD Stress Index |
|  | Quality of life | IBDQ score | IBDQ |
| Selby 2007 | Quality of life | Change in Assessment of Quality of Life score | Assessment of Quality of Life Questionnaire |
|  | Quality of life | Change in IBDQ from baseline | IBDQ |
|  | Quality of life | Change in SF-36 score | SF-36 |
| Feagan 2008 | Quality of life | Change in SF-36 from baseline | SF-36 |
| Mantzaris 2009 | Quality of life | Change in IBDQ from baseline | IBDQ |
| Rossi 2009 | Quality of life | Change in IBDQ from baseline | IBDQ |
| Takagi 2009 | Quality of life | Mean IBDQ score | IBDQ |
| Valentine 2009 | Quality of life | Change in IBDQ from baseline | IBDQ |
|  | Quality of life | Change in SF-36 score | SF-36 |
|  | Quality of life | Change in VAS score | EuroQOL-derived visual analogue scale |
| Holtmeier 2011 | Quality of life | Change in IBDQ from baseline | IBDQ |
| Watanabe 2012 | Quality of life | Change in SF-36 mental component summary score from baseline | SF-36 MCS |
|  | Quality of life | Change in SF-36 physical component summary score from baseline | SF-36 |
|  | Quality of life | IBDQ score | IBDQ |
| Keefer 2012 | Quality of life | Mean IBDQ score | IBDQ |
|  | Quality of life | Mean IBD-SES score | IBD-SES |
|  | Quality of life | Mean PSQ-R score | PSQ-R |
|  | Treatment compliance | Mean MAS score | Medical Adherence Scale |
| Feagan 2014 | Quality of life | Change in SF-36 from baseline | SF-36 |
| Piche 2014 | Bowel symptoms | IBS severity scoring system | IBS severity scoring system |
|  | Quality of life | BDI severity of depression score | BDI |
|  | Quality of life | FIS severity of fatigue score | Fatigue Impact Scale |
|  | Quality of life | HAD severity of anxiety score | Hospital Anxiety and Depression Scale |
|  | Quality of life | IBDQ score | IBDQ |
| Wenzl 2015 | Quality of life | IBDQ score | IBDQ |
| **Maintenance studies of surgically induced remission (n=4)** | | | |
| Ewe 1999 | Quality of life | Patient global assessment of wellbeing | Patient Global Assessment |
| Reinisch 2010 | Quality of life | Mean IBDQ change from baseline | IBDQ |
| Savarino 2013 | Quality of life | Mean IBDQ score | IBDQ |
| Fedorak 2015 | Quality of life | Mean IBDQ score | IBDQ |
| Note: IBDQ, Inflammatory Bowel Disease Questionnaire; SF-36, Short-Form 36; SF-36 MCS, SF-36 mental component summary score; SF-36 PCS, SF-36 physical component summary score; SF-12, Short-Form 12; GIQLI, Gastrointestinal Quality of Life Index; STAI-X2 State trait Anxiety Inventory instrument; PSKB, Psychiatric and Socio-communicative finding standardised clinical interview; BDI, Beck's Depression Inventory instrument; IBD-SES, Inflammatory Bowel Disease Self-Efficacy Scale; PSQ-R, Perceived Stress Questionnaire-Recent  ** Trials in fistula patients | | | |
